# The Caribbean intertidal mite *Alismobates inexpectatus* (Acari, Oribatida), an unexpected case of cryptic diversity?

**DOI:** 10.1007/s13127-023-00624-9

**Published:** 2023-09-29

**Authors:** Tobias Pfingstl, Iris Bardel-Kahr, Sylvia Schäffer

**Affiliations:** Institute of Biology, Karl-Franzens-University Graz, Universitätsplatz 2, 8010 Graz, Austria

**Keywords:** Gulf stream, Central America, Antilles, Morphometry, Haplotype

## Abstract

Molecular genetic analyses of Caribbean populations of the supposedly widespread intertidal oribatid mite *Alismobates inexpectatus* revealed the existence of a cryptic species. The new species, *Alismobates piratus* sp. n., shows considerable *COI* and *18S* rRNA gene sequence divergences and although morphometric analyses indicate considerable variation between the taxa, no distinguishing morphological feature could be detected. The extreme intertidal environment is suggested to be responsible for the observed morphological stasis of the two species and vicariance is supposed to be responsible for their speciation. *Alismobates piratus* sp. n. was found on Hispaniola, Guadeloupe, Barbados and Curaçao indicating a predominant distribution on the Greater and Lesser Antilles, whereas the occurrence of *A. inexpectatus* is primarily restricted to Central America, the northern Caribbean and the Greater Antilles. Haplotype network analyses indicate distinct geographic structuring and the absence of recent gene flow among the Antillean *A. piratus* sp. n. populations. Central American and Antillean populations of *A. inexpectatus* show similar patterns but populations from Bermuda and the Bahamas are characterized by a common origin and subsequent expansion. Genetic landscape analysis demonstrates that vast stretches of open ocean, like the Caribbean Basin and the Western Atlantic, act as rather effective barriers, whereas the continuous continental coastline of Central and North America may facilitate dispersal. Genetic data also indicates that the Gulf Stream plays an important role for the biogeography of intertidal oribatid mites as it may be responsible for the strong link between Central and North American populations as well as for the colonization of Bermuda.

**Supplementary Information:**

The online version contains supplementary material available at 10.1007/s13127-023-00624-9.

## Introduction

Intertidal oribatid mites represent a small group of tiny arachnids that have adapted to the littoral environment. There, they live in the zone between low and high tide using diverse algae as substrate and food source (e.g. Pfingstl, 2017). For a long time, Caribbean coasts seemed to be devoid of these organisms, but a recent study (Pfingstl, 2021) revealed wide distributions of members of two families, the Fortuyniidae and the Selenoribatidae, and demonstrated that these mites are common parts of the littoral Caribbean fauna. The Caribbean region consists of continental fragments, namely the Greater Antilles, which broke off the mainland ca. 40 million years ago, and of recent volcanic islands, the Lesser Antilles which have emerged in the last 10 million years (Iturralde-Vinent & MacPhee, 1999). The geological history of this area has created unique conditions for colonization and diversification and thus features a tremendous biodiversity and high levels of endemism (e.g. Ricklefs & Bermingham, 2008).

Mites are very small arthropods with limited dispersal abilities and therefore widespread species, especially in the Caribbean with its numerous islands separated by oceanic barriers, would be rather exceptional. Indeed, several intertidal oribatid species, with supposedly wide distributions across the Caribbean, recently turned out to be subject to high cryptic diversity or to represent cryptic species complexes (Pfingstl et al., 2019a, b, 2021, 2022). Cryptic species are defined as species that are classified as a single nominal species because they are at least superficially anatomically identical (Bickford et al., 2007). This means that the morphology of theses taxa has remained stable and is highly conserved, whereas they have strongly diversified on a genetic level due to a lack of gene flow. For example, different members of the cryptically diverse *Carinozetes mangrovi* Pfingstl et al., 2014 a selenoribatid Caribbean intertidal mite, cannot be distinguished from each other based on morphology, but actually consist of three distinct genetic lineages, a Northern, an Antillean and a Pacific lineage (Pfingstl et al., 2019a, b). Similarly, Caribbean members of the fortuyniid intertidal *Fortuynia atlantica* Krisper & Schuster, 2008 show an identical morphology throughout their distribution range, but in fact include a genetically distinct species occurring on the Lesser Antilles (Pfingstl et al., 2022). An even more intriguing case is that of the Caribbean *Thalassozetes barbara* Pfingstl, 2013, another selenoribatid mite, that could be demonstrated to comprise seven phenotypically almost identical but genetically distinct species, with nearly every species being an island or a short-range endemic (Pfingstl et al., 2021). Although the extent of diversity and the cause for it may differ between the cases, the found morphological stasis of all is supposed to be a result of stabilizing selection caused by the extreme conditions of the intertidal environment (Pfingstl et al., 2019a, b, 2021, 2022).

Another known Caribbean species is the fortuyniid *Alismobates inexpectatus* Pfingstl & Schuster, 2012, which was first discovered on the small Archipelago of Bermuda in the Western Atlantic (Pfingstl & Schuster, 2012). At the time, it was also the first and thus unexpected record of this genus from the Atlantic area, therefore the name *inexpectatus* was given to this species. Presently, *A. inexpectatus* is known to occur on the shores of Florida, the Bahamas, Hispanola, Central America and several islands of the Lesser Antilles (Pfingstl, 2021), and thus shows apparently a trans-Caribbean distribution. However, in view of the above-mentioned circumstances, a wide-spread single species *Alismobates inexpectatus* seems to represent an unlikely hypothesis and finding cryptic diversity within this taxon would not be unexpected at all.

To screen members of *A. inexpectatus* for cryptic diversity, we investigated more than 500 specimens from nine different regions/islands of the Caribbean and the Western Atlantic using morphometric and molecular genetic analyses. Our specific aims were (I) to reveal cryptic taxa if present, (II) to assess morphological and genetic variation among different *A. inexpectatus* populations, and (III) to interpret found patterns from a bio- and paleogeographic point of view.

## Material and methods

### Sample collection and locations

In the years 2016–2018, coastal mites were collected during three fieldtrips to Bermuda and several regions in the Caribbean. Samples of intertidal algae were scraped off rocks or mangrove roots with a knife during low tide. Algae were then put in Berlese-Tullgren funnels for about 24 h to extract living mites. Specimens were preserved and stored in absolute ethanol for subsequent morphological and molecular genetic investigation. All samples were taken by T. Pfingstl and A. Lienhard, except for the sample from Costa Rica, which was collected by G. Kunz. Details are given in Table 1.

**Table 1:** Details about samples taken in the years 2016–2018. The abbreviation B.o.r means *Bostrychia* (intertidal alga) on rock. If two labels are given for one location, then two separate samples were taken in more or less the same spot

country/island	location	label	coordinates	habitat	date
Bermuda	Whalebone Bay	BD_07, 08	32.365424, -64.713527	*Gardnerula* on rock	23 May 2018
	Whalebone Bay	BD_36	32.365424, -64.713527	*Gardnerula* on rock	28 May 2018
	Tobacco Bay	BD_11	32.389109, -64.678894	B.o.r	24 May 2018
	Hungry Bay	BD_21	32.290249, -64.759893	B.o.r	26 May 2018
	Coney Island	BD_25	32.357888, -64.716516	brown short algae on rock	26 May 2018
	Cooper's Island	BD_40	32.346514, -64.652784	B.o.r	28 May 2018
Bahamas	Paradise Island	BH_03	25.085983, -77.299663	B.o.r	18 Feb. 2017
	Paradise Island	BH_25	25.085983, -77.299663	B.o.r	22 Feb. 2017
	Love Beach	BH_08	25.062984, -77.491670	B.o.r	19 Feb. 2017
	Compass Point	BH_10	25.065252, -77.470981	B.o.r	19 Feb. 2017
	South Beach	BH_16, 17	25.001339, -77.350229	B.o.r	20 Feb. 2017
	Jaws Beach	BH_19	25.018155, -77.546636	*Gardnerula* on rock	21 Feb 2017
	Montagu beach	BH_21	25.073943, -77.307127	*Bostrychia* on mangrove	22 Feb. 2017
Barbados	Bathsheba	BA_13	13.213011, -59.520318	B.o.r	25 Feb. 2017
	Bathsheba	BA_15	13.21393, -59.521825	B.o.r	25 Feb. 2017
	Bathsheba	BA_28, 30	13.062537, -59.541903	B.o.r	28 Feb. 2017
	Oistins	BA_22	13.062537, -59.541903	diverse algae on rocks	27 Feb. 2017
Costa Rica	Manzanillo	CR_01	9.683929, -85.202774	diverse algae on rocks	12 Feb. 2018
Curaçao	Boca Ascención	CU_15	12.273242, -69.052882	B.o.r	5 Feb. 2016
Dominican Rep	Boca Chica	DR_03	18.447819, -69.620430	B.o.r	8 Feb. 2016
	Samaná	DR_10	19.201034, -69.324964	B.o.r	11 Feb. 2016
Florida U.S.A	Indian Key Fill	FL_12	24.892094, -80.670667	B.o.r	13 Feb. 2017
	Sombrero Beach	FL_16	24.691913, -81.083700	B.o.r	13 Feb. 2017
	Islamorada	FL_18	24.9378169, -80.612182	B.o.r	13 Feb. 2017
	Key Largo	FL_19	25.183048, -80.353081	diverse algae on rocks	13 Feb. 2017
Guadeloupe	Capesterre Belle-Eau	GU_09	16.034611, -61.564938	B.o.r	19 Feb. 2016
Panamá	Isla Colon	PA_39	9.385454, -82.23524	diverse algae on rocks	7 Feb. 2017
	Isla Bastimentos	PA_41	9.344917, -82.180404	diverse algae on boulder	8 Feb. 2017

### Molecular genetics

Specimens from Costa Rica were not used for this study, because we had no permit to perform molecular genetic analyses with this collected material.

For genetic analyses, whole genomic DNA was extracted from all 238 specimens included in this study using Chelex resin (Pfingstl et al., 2022). Region 2 of the mitochondrial cytochrome c oxidase subunit 1 gene (*COI*) was amplified with primers by Otto and Wilson (2001), these are: Mite COI – 2F TTY GAY CCI DYI GGR GGA GGA GAT CC and Mite COI – 2R GGR TAR TCW GAR TAW CGN CGW GGT AT. The nuclear *18S* rRNA gene (*18S*) was amplified using the primers designed by Dabert et al. (2010). PCR amplification with subsequent DNA purification and cycle sequencing were conducted as described in Pfingstl et al. (2022), with the exception of the second purification step using the BigDye XTerminator Purification Kit according to the protocol by Applied Biosystems. Automatic capillary sequencing and sequence visualization then was conducted on an ABI3500XL (Applied Biosystems) device.

All generated sequences are available from GenBank under the accession numbers OR358561 to OR358798 for *COI* and OR360272 to OR360328 for *18S* rRNA (see Appendix).

We sequenced 622 bp of the *COI* gene in all 238 specimens and 1,806 bp of the *18S* rRNA gene in 57 individuals, which comprised representatives of all potential mitochondrial lineages. Additionally, sequences of nine species belonging to the superfamily Ameronothroidea, a group of mainly marine associated oribatid mites, were taken from GenBank and added to the *COI* and *18S* rRNA alignments (see Appendix), whereof two *Fortuynia* species were chosen as outgroup taxa. In a first trial, we analyzed both genes separately and in a next step, fragments of both genes were combined for 63 specimens (only those with all available sequences were used) in a concatenated dataset with a final length of 2445 bp.

PhyloSuite v.1.2.2 (Zhang et al., 2020) was used to perform all phylogenetic analyses. In *COI* and the concatenated dataset, PartionFinder2 (Lanfear et al., 2017) was applied to find the best partitioning scheme and evolutionary model under greedy algorithm. Sequences of *18S* rRNA gene were aligned with MAFFT (Katoh & Standley, 2013) using parameters '–auto' strategy and normal alignment mode. Gap sites were removed with trimAl (Capella‐Gutiérrez et al., 2009) using automated1 command. Maximum Likelihood (ML) phylogenies were inferred using IQ-tree (Nguyen et al., 2015) and Bayesian inference (BI) phylogenies using MrBayes 3.2.6 (Ronquist et al., 2012), both programs provided on the platform PhyloSuite. ML was performed under edge-linked partition model in *COI* and HKY + I + F model in *18S* rRNA for 5000 ultrafast bootstraps (Minh et al., 2013), as well as the Shimodaira–Hasegawa–like approximate likelihood-ratio test (Guindon et al., 2010). BI phylogenies were inferred under the same models as ML, performing two parallel runs with four chains, each for 20 million generations sampling every 2,500 generation and discarding the first 25% as burn-in.

Dataset for *COI* haplotype networks contained only *Alismobates* specimens. The TCS networks (Clement et al., 2002) were reconstructed using PopArt (Leigh & Bryant, 2015).

For species delimitation analyses (SDA) based on *COI* data, we employed two methods: the “Assemble Species by Automatic Partitioning” (ASAP) method (Puillandre et al., 2021) and the ML partition “Bayesian Poison Tree Process “ (bPTP-ML) model (Zhang et al., 2013). The applied settings and programs/packages for the SDA analyses followed Schäffer and Koblmüller (2020), except for ASAP applying default parameters and uncorrected p-distances. To calculate Rodrigo´s P(Randomly Distinct) (*P*_(*R*D)_) measures, we used the species delimitation plugin (Masters et al., 2011) in Geneious Prime 2023.1.2 (https://www.geneious.com).

To assess correlation between geographical and genetic distances, a Mantel test with 10,000 permutations was performed using the program Alleles In Space (AIS) (Miller, 2005). In addition, we analyzed the genetic landscape shape, which allows the visualization of genetic distance patterns across the sampling area (Miller, 2005). The three-dimensional plots produced by AIS were illustrated from different angles.

Nucleotide diagnosis for the herein investigated *Alismobates* species was applied using the package Spider (Brown et al., 2012) in R (R Core Team, 2022). We used same alignments of the respective fragment as in the analyses above, with the exception that the *Fortuynia* species (outgroup) were excluded.

### Morphometric study

For the morphometric study, specimens were placed in lactic acid (temporary slides) and measurements were performed using a compound light microscope (Olympus BH-2) and ocular micrometer. Individuals used for morphometric investigations were not the same as used for molecular genetic studies but belonged to the exact same populations (patch of algae ca. 10 cm^2^).

**Fig. 1:**
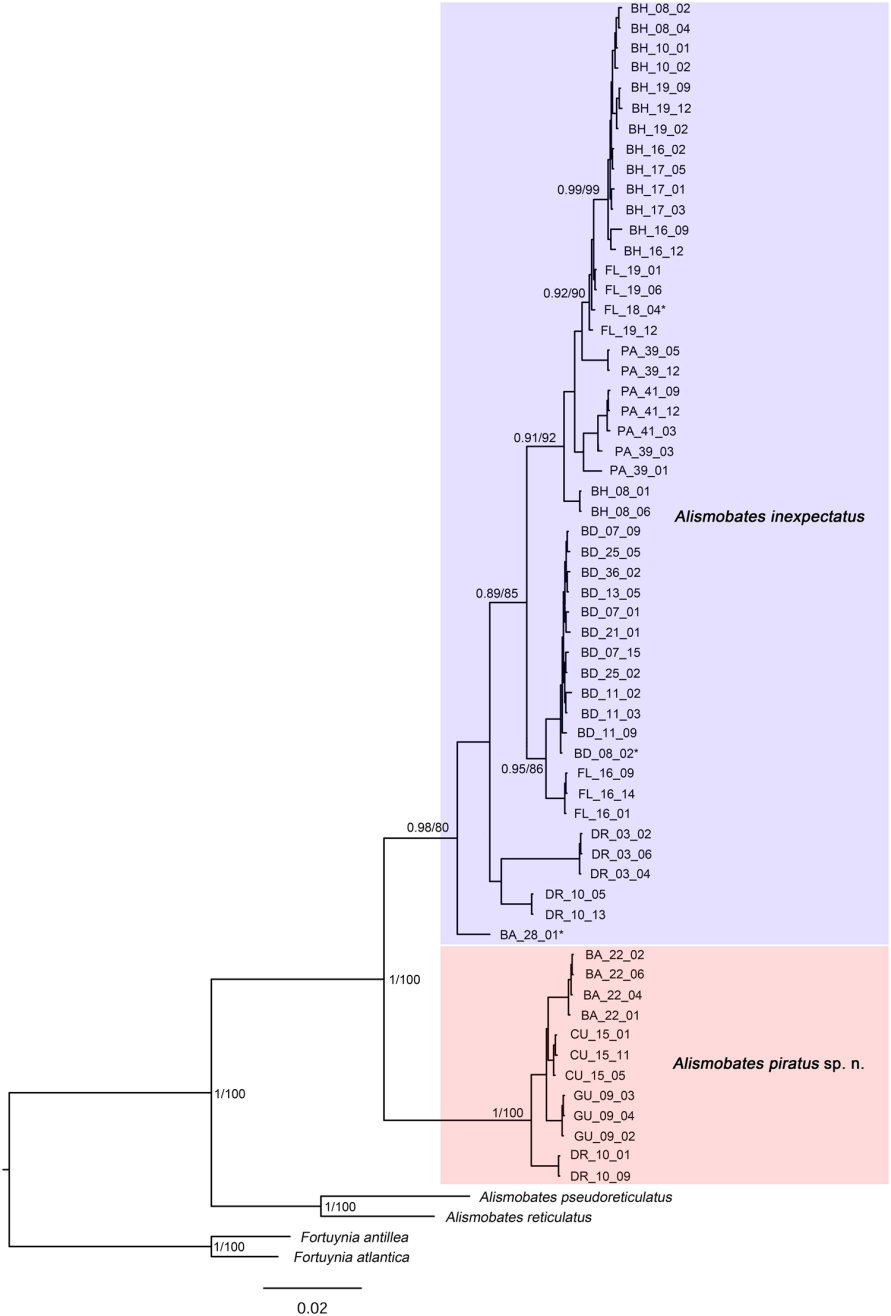
Maximum likelihood tree (IQ-tree) inferred from a concatenated dataset of *COI* and *18S* rRNA gene fragments. Numbers at nodes represent Bayesian posterior probability values (shown > 0.85) and bootstrap values (shown > 80) for ML. Sequences marked with an * were taken from GenBank

A set of 16 continuous variables (Pfingstl & Baumann, 2017; Fig. 1a, b, p.118) was measured in 271 specimens of *Alismobates* from nine different Caribbean regions; Bermuda: 36 specimens (BD_07, BD_21); Bahamas: 76 specimens (BH_03, BH_08, BH_16, BH_19, BH_21, BH_25); Barbados: 19 specimens (BA_15, BA_22, BA_30); Costa Rica: 20 specimens (CR_01); Curaçao: 14 specimens (CU_15); Dominican Republic: 18 specimens (DR_03, DR_10); Florida: 44 specimens (FL_12, FL_16, FL_19); Guadeloupe: 5 specimens (GU_09); Panama: 39 specimens (PA_39, PA_41). [Bermuda is part of the Western Atlantic and not the Caribbean, but for reasons of simplicity it is listed here and in the following parts under Caribbean or Northern Caribbean.]

For univariate comparisons, mean, standard deviation, minimum and maximum of each variable were calculated. To compare the means of the variables between species a Kruskal–Wallis test was performed and for comparison between populations within each species a Kruskal–Wallis and a Mann–Whitney-U test were conducted.

Multivariate analyses investigating differences between putative species and geographic groups respectively, included a Principal Component Analysis (PCA), Non-metric Multidimensional Scaling (NMDS; based on Euclidian distances, two-dimensional) and Discriminant Analysis (LDA); all analyses were performed on log_10_-transformed raw and size-corrected data. No rotation was applied to the multivariate data. Size correction was done by dividing each variable through the geometric mean of the respective specimen. For species discrimination all populations from a species were pooled, and populations of *A. inexpectatus* were pooled according to specific geographic areas for assessing intraspecific variation (e.g. populations from Panama and Costa Rica were pooled under ‘Central America’). All analyses were performed with PAST version 3.11 (Hammer et al., 2001).

### Drawings and photographs

Specimens were embedded in Berlese mountant for microscopic investigation in transmitted light and these preparations were studied and depicted using an Olympus BH-2 Microscope. Drawings were first scanned, then processed and digitized with the free and open-source vector graphics editor Inkscape (https://inkscape.org).

For photographic documentation, specimens were air-dried and photographed using a Keyence VHX-5000 digital microscope with automated image stacking.

## Results

### Molecular genetic results

Molecular genetic analyses of mitochondrial *COI* and nuclear *18S* rRNA gene sequences clearly highlighted the presence of two different species. Uncorrected p-distances between these two species were 14.8% in the *COI* gene and 0.2% in the *18S* rRNA gene. Intraspecific mean distances ranged from 2.7 to 5.3% in *COI* (Table 2) and were 0% in the *18S* rRNA. Thus, a barcoding gap was evident in both markers. Furthermore, both species clustered in highly supported, distinct clades in the ML and BI phylogenies of the single gene analyses, as well as the concatenated dataset (Fig. 1 and Suppl. Figures). Single-locus delimitation based on COI revealed 12 to 15 putative species (excluding outgroups): ASAP shows the best support for a total of 12 putative *Alismobates* species with an ASAP-score of 2.00. The partition with only two Caribbean *Alismobates* species results in a score of 11.50 and thus significantly shows weaker support. A similar picture is given by the bPTP model with 15 well to highly supported, putative Caribbean *Alismobates* species (Suppl. Fig. S5).

**Table 2:** Mean uncorrected pairwise distances (1) between and (2) within *Alismobates* (A*.)* and *Fortuynia* (*F*.) species. Values for *COI* gene are given in the left lower corner and for *18S* rRNA in the right upper corner. Standard deviation values shown in brackets. n/c = not possible to estimate evolutionary distances

	**Pairwise distances between species**						**Pairwise distances within species**	
	*inexpectatus*	*piratus* n.sp*.*	*A* *pseudoreticulatus*	*A* *reticulatus*	*F* *atlantica*	*F* *antillea*	*COI-2*	*18S*
A. *A. inexpectatus*		0.002 (0.001)	0.016 (0.003)	0.014 (0.003)	0.029 0.014 (0.004)	0.029 0.014 (0.004)	0.053 (0.005)	0
A. *A. piratus* n.sp.	0.148 (0.012)		0.018 0.014 (0.003)	0.016 0.014 (0.003)	0.030 0.014 (0.004)	0.030 0.014 (0.004)	0.027 (0.003)	0
A. *A. pseudoreticulatus*	0.158 (0.015)	0.148 (0.015)		0.003 0.014 (0.001)	0.029 0.014 (0.004)	0.030 0.014 (0.004)	0.007 (0.003)	n/c
A. *A. reticulatus*	0.156 (0.014)	0.153 (0.015)	0.149 (0.016)		0.027 0.014 (0.004)	0.028 0.014 (0.004)	n/c	n/c
*F. atlantica*	0.147 (0.013)	0.175 (0.015)	0.154 (0.015)	0.165 (0.015)		0.001 0.014 (0.001)	0.061 (0.010)	0
*F. antillae*	0.160 (0.014)	0.175 (0.016)	0.149 (0.015)	0.177 (0.017)	0.097 (0.011)		n/c	n/c

According to the presented results, the newly discovered lineage was given species rank and will be referred to as *Alismobates piratus* sp. n. The calculated Randomly Distinct PRD value for *Alismobates piratus* sp.n. was 0.05 and is thus inconclusive; the value for *A. inexpectatus* was 0.09 and indicates that its structure follows the coalescent model and does not contain any further cryptic species.

**Fig. 2:**
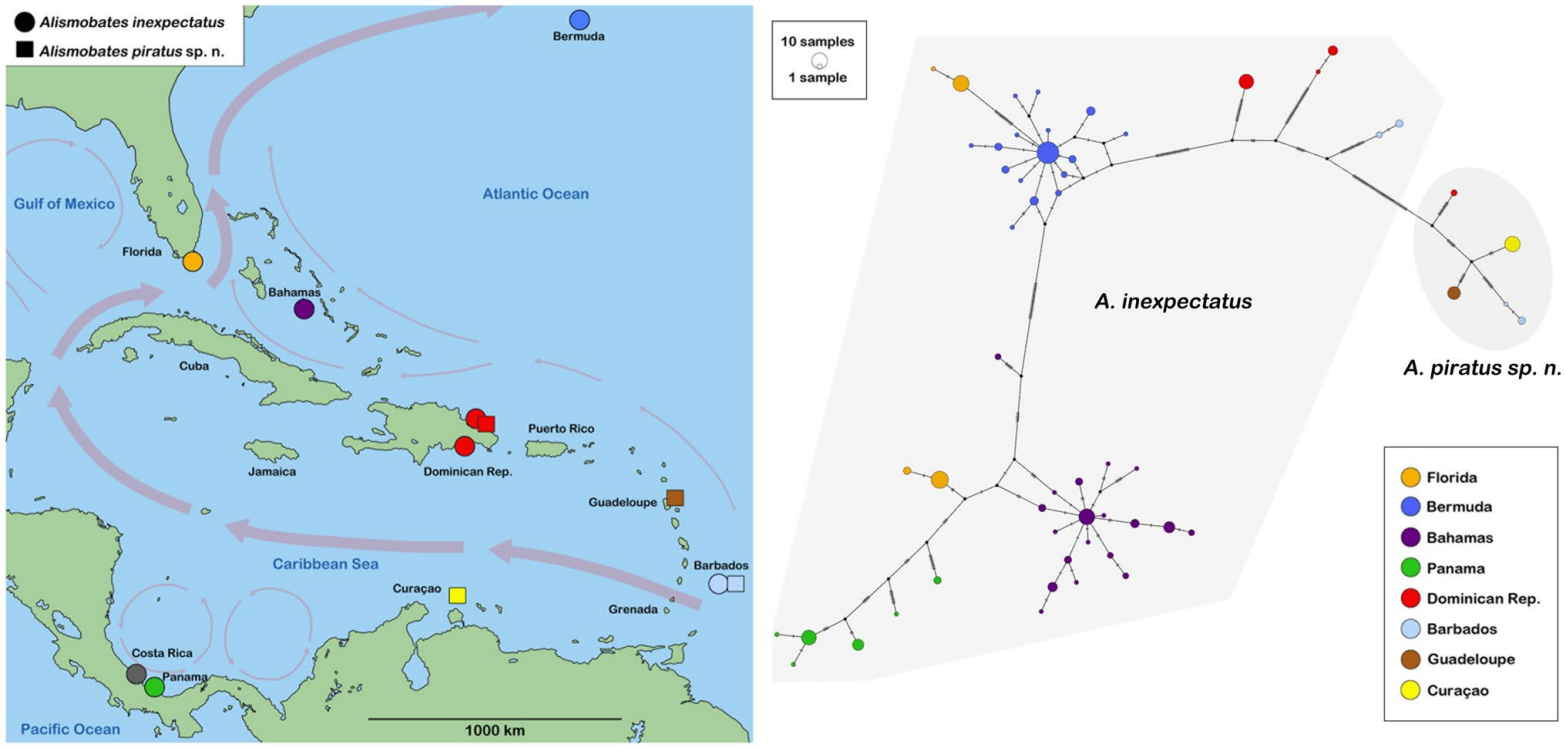
Map showing records of the two *Alismobates* species in the Caribbean and TCS haplotype network based on their *COI* sequences. Light red arrows on map indicate ocean currents and their directions. Each circle in the network corresponds to one haplotype and its size is proportional to its frequency, the number of mutations is indicated as hatch marks. Small black circles represent intermediate haplotypes not present in the dataset. Colors refer to different sample locations grey shades represent the different species

The *COI* haplotype diversity in the new *Alismobates piratus* sp. n. was relatively low with only four different haplotypes which, however, were distinctly separated from each other (Fig. 2).

**Fig. 3:**
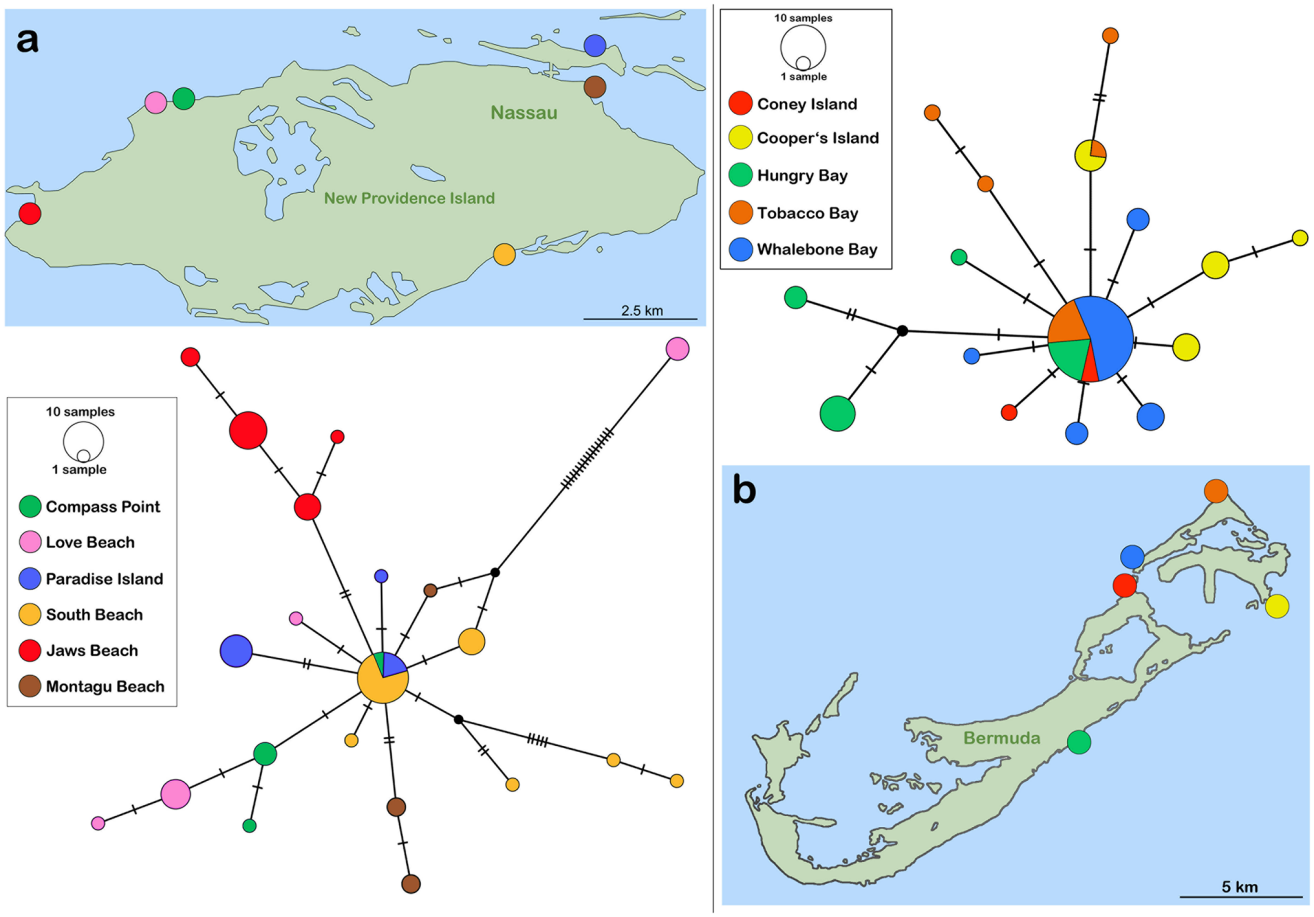
Haplotype networks based on *COI* sequences of *A. inexpectatus* populations from the Bahamas and Bermuda. Each circle corresponds to one haplotype and its size is proportional to its frequency. The number of mutations is indicated as hatch marks. Small black circles represent intermediate haplotypes not present in the dataset. Colors refer to different locations as indicated on the respective map. **a**) New Providence Island, Bahamas. **b**) Bermuda

Haplotype diversity was higher in *Alismobates inexpectatus* with a total of 53 haplotypes. Specimens from Central America and the Antilles showed six and five haplotypes, respectively, with distinct structuring, whereas the Northern Caribbean group was with 42 haplotypes the most diverse one (Fig. 2). In the case of individuals from Bahamas, respectively, Bermuda, the haplotype networks revealed no geographic sub-structuring (Fig. 3).

**Fig. 4:**
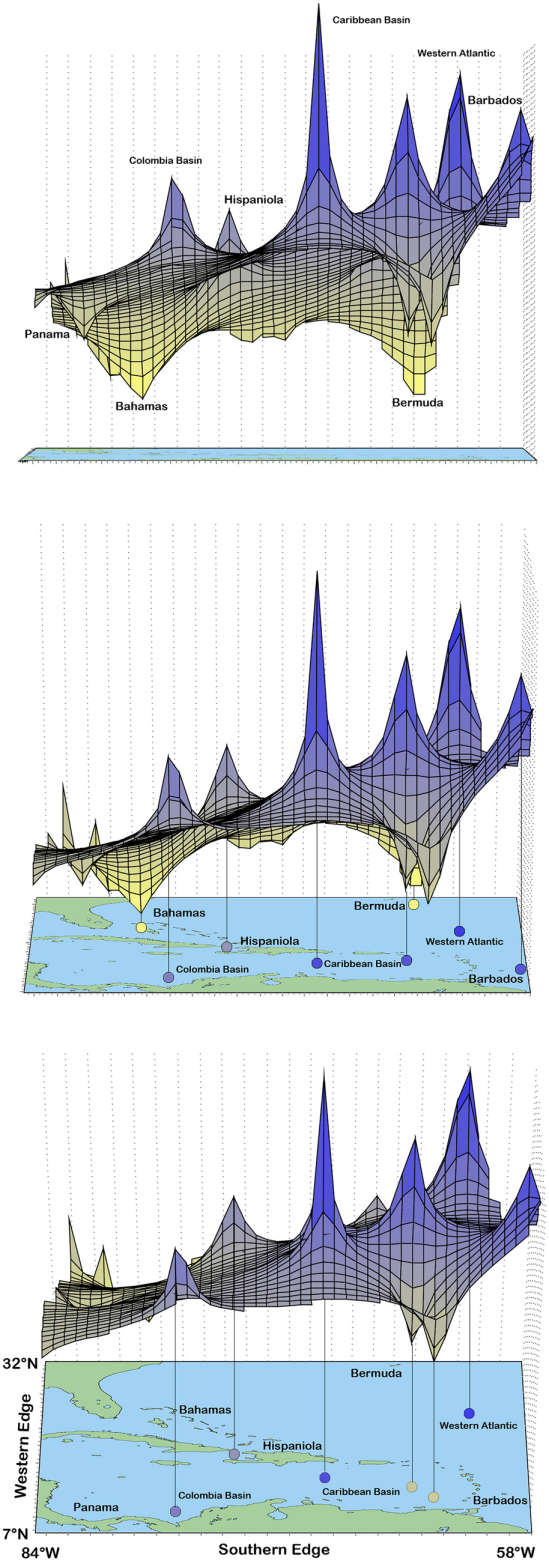
Multidimensional graph produced by the genetic landscape shape interpolation analysis, shown from different angles. Dark blue peaks represent areas with high genetic discontinuities and yellow valleys indicate low genetic distances of individuals across the distribution of *Alismobates* species in the Caribbean. Most important peaks and valleys are indicated on the map below

Genetic landscape interpolation analysis at individual level of both species qualitatively supported the results obtained from the haplotype networks. It showed basically lower genetic distances in the Northern Caribbean and Central America suggesting more connectivity between these two large areas (Fig. 4). High genetic differentiation among individuals became evident in the Western Atlantic, the Caribbean and Colombian Basin as well as in Hispaniola and Barbados.

**Fig. 5:**
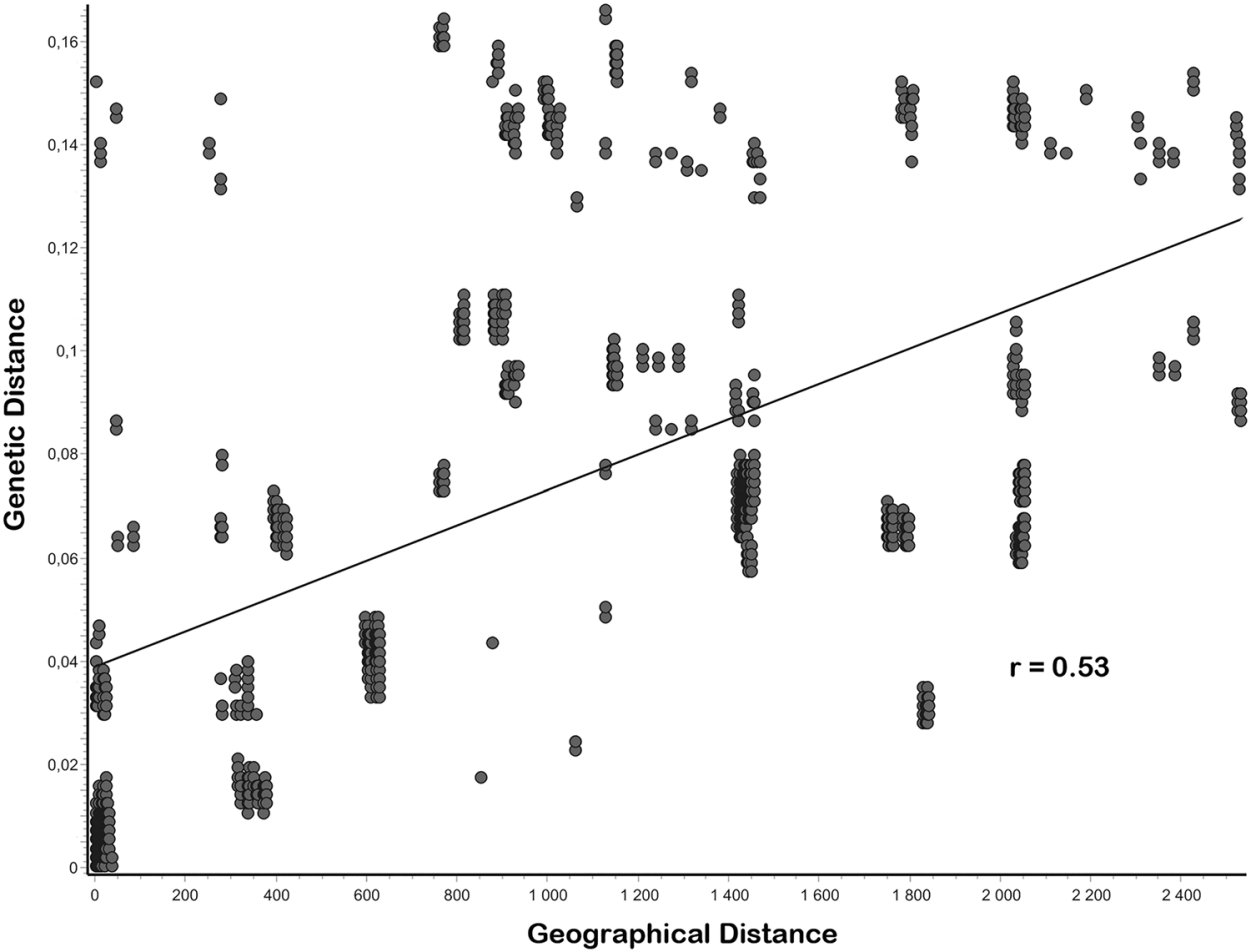
Mantel test for *COI* data of Caribbean *Alismobates* species with correlation coefficient (r), showing the correlation of genetic and geographic distances

Results of the Mantel test calculated in AIS revealed a low but statistically significant correlation between geographical and genetic distances (r = 0.53, *P* < 0.001) indicating that genetic distances can be explained in part by geographic distances (Fig. 5).

## Description of new species

Family Fortuyniidae Hammen, 1963.

Genus *Alismobates* Luxton, 1992.

***Alismobates piratus* sp. nov.** urn:lsid:zoobank.org:act: 35A22D71-5078-4D1A-AA61-A6DCD5A8E7E5.

Type material: Holotype male (size 356 µm x 240 µm), Curaçao: Boca Ascención (CU_15) *Bostrychia* on rocks in the upper eulittoral area, 5 Feb. 2016; paratype (1 female 375 µm x 249 µm) same data as for holotype, both types deposited in the Senckenberg Museum für Naturkunde Görlitz (SMNG).

Etymology: The species occurs in the Caribbean area which is well known for its long historical era of piracy. The specific epithet ‘*piratus’* refers to the Latin word for pirate (‘pirata’) and is given as noun in apposition.

Species diagnosis: Dark brown sclerotized mites. Average length 362 μm, mean width 233 μm. Notogaster oval in shape. No conspicuous sexual dimorphism; there is only the common sexual dimorphism in overall body size, with females being generally slightly larger. Van der Hammen’s Organ well developed, typical for the genus. Sensillum clavate, spinose. One pair of large cuticular ridges in position of prodorsal lamellae. Interlamellar setae minute. Lenticulus (light spot) large, variable in shape and with irregular borders. Areas flanking lenticulus conspicuously granular. Fourteen pairs of short and simple, notogastral setae, associated with small porose areas. Epimeral setation 3–1-2–2. Four pairs of genital setae. One pair of aggenital setae. Three pairs of adanal setae. Two pairs of anal setae. Legs monodactylous with large claw. Porose areas on trochanters III and IV and all femora. Leg setation (chaetome, solenidia): Leg I 0–4-2–3-18, 1–2-2; leg II 0–4-2–3-15, 1–1-1; leg III 1–3-1–3-15, 1–1-0; leg IV 1–2-2–3-12, 0–1-0.

Diagnostic nucleotides (only unique diagnostic characters are given): In *COI*, position 7 is occupied by base G, position 17 by base T, position 19 by base A, position 73 by base T, position 82 by base C, position 133 by base T, position 199 by base T, position 340 by base A, position 346 by base A, position 391 by base G, position 409 by base C, position 538 by base C, position 551 by base G, position 574 by base C, position 608 by base C and position 613 by base T.

In *18S* rRNA, position 728 is occupied by base G, position 768 by base A and position 1350 by base A.

Distribution: Presently, *Alismobates piratus* sp. n. is known to show a distribution range from Hispanola to the Lesser Antilles. There are reports from the Lesser Antillean islands of Guadeloupe, Barbados and Curaçao, and there is a record from the northeastern coast of the Dominican Republic, namely from shores of the Samaná Peninsula (see Fig. 2). Distributions on Barbados and the Dominican Republic coincide with occurrences of *A. inexpectatus* whereas specimens of both species were found syntopically (in a single sample of ca. 10cm^2^ algae) on the coast of Samaná.

**Fig. 6:**
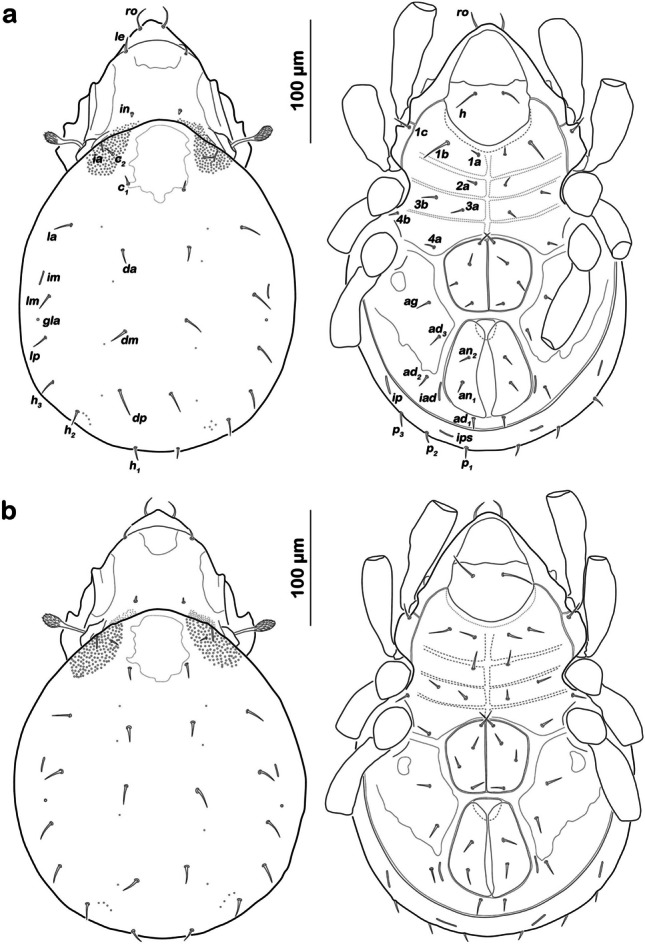
Dorsal (left) and ventral (right) depictions (legs omitted or only partially drawn) of the two genetically distinct *Alismobates* species, illustrating the phenotypic similarity. **a**) *A. piratus* sp. n. (specimen from Barbados BA_22); **b**) *A. inexpectatus* (specimen from the Dominican Republic DR_03)

Remarks: The new species *A. piratus* sp. nov. is morphologically identical to *A. inexpectatus* (Fig. 6) and thus cannot be distinguished from it based on microscopic investigation only.

The lengths and shapes of certain characters, i.e. sensillum and body setae, may look slightly different in some of our depictions, but these are results of slightly different observational perspectives or of different orientations of these characters. These structures are bendable and they are inserted on a convex surface, therefore a slightly different viewing angle may result in a divergent appearance. For these reasons, it is also extremely difficult to make standardized measurements of these traits. However, we observed more than 270 specimens under the microscope and could not find any reliable diagnostic character separating the two species (to the best of our knowledge), therefore, we herein provide the species diagnosis only, which is also valid for *A. inexpectatus*; a detailed description applying to both species is given in Pfingstl and Schuster (2012). Due to the identical phenotypes, species determination should always include the analysis of molecular genetic markers.

### Morphometric results

#### Interspecific variation

Comparing the two species with univariate statistics, size ranges show overlaps in each variable but mean values are generally higher in *Alismobates inexpectatus* indicating a trend towards a larger body size in this species. Kruskal–Wallis test found significant differences in all measured morphological variables except for the anterior notogastral width *nwc*_*1*_ (Table 3).

**Table 3:** Univariate statistics and comparison of the two Caribbean *Alismobates* species. Minimum–maximum (mean ± standard deviation) of each measured variable given in µm. Results of Kruskal–Wallis Test (KW) are given; * = p < 0.05, ** = p < 0.01, *** = p < 0.001

	*Alismobates piratus sp. n* (n = 39)	*Alismobates inexpectatus* (n = 232)	KW
*bl*	320–391 (362 ± 18.23)	338–406 (371 ± 15.34)	*
*dpc*	95–108 (101 ± 3.62)	99–120 (110 ± 3.93)	***
*dPtI*	145–163 (153 ± 4.36)	145–175 (163 ± 5.15)	***
*db*	92–109 (101 ± 4.34)	92–117 (105 ± 4.29)	***
*ll*	52–99 (81 ± 8.99)	52–92 (71 ± 7.31)	***
*nwc*_*1*_	166–212 (189 ± 11.76)	157–225 (189 ± 12.87)	-
*nw*_*da*_	206–255 (233 ± 11.54)	200–274 (241 ± 14.2)	**
*nw*_*dm*_	200–255 (230 ± 12.63)	200–274 (242 ± 14.09)	***
*cl*	92–108 (102 ± 4.05)	91–111 (100 ± 4.42)	*
*cw*	71–77 (75 ± 1.99)	71–86 (76 ± 2.42)	***
*dcg*	71–89 (78 ± 4.68)	71–92 (83 ± 4.18)	***
*dac3*	120–135 (128 ± 3.92)	114–151 (135 ± 4.44)	***
*gl*	49–68 (58 ± 6.04)	49–71 (60 ± 5.71)	**
*gw*	63–81 (72 ± 5.96)	65–89 (77 ± 6.43)	***
*al*	74–95 (86 ± 5.62)	78–102 (88 ± 4.15)	**
*aw*	68–80 (74 ± 3.03)	68–95 (79 ± 4.45)	***

Multivariate statistics – PCA based on raw data results in a clustering of both species with a small overlap, with the first three components accounting for 79.59% of total variation (PC1 53.52, PC2 19.97, PC3 6.1). Highest loadings on PC1 are shown by characters of the genital opening, namely *gl* and *gw*. PCA on size corrected data resulted in a clustering of both species with a considerable overlap and the first three components accounting for 66.48% of total variation (PC1 32.69, PC2 21.71, PC3 12.08). Highest loadings are shown by the variables *ll*, *nwc*_*1*_ and *nwdm*.

**Fig. 7:**
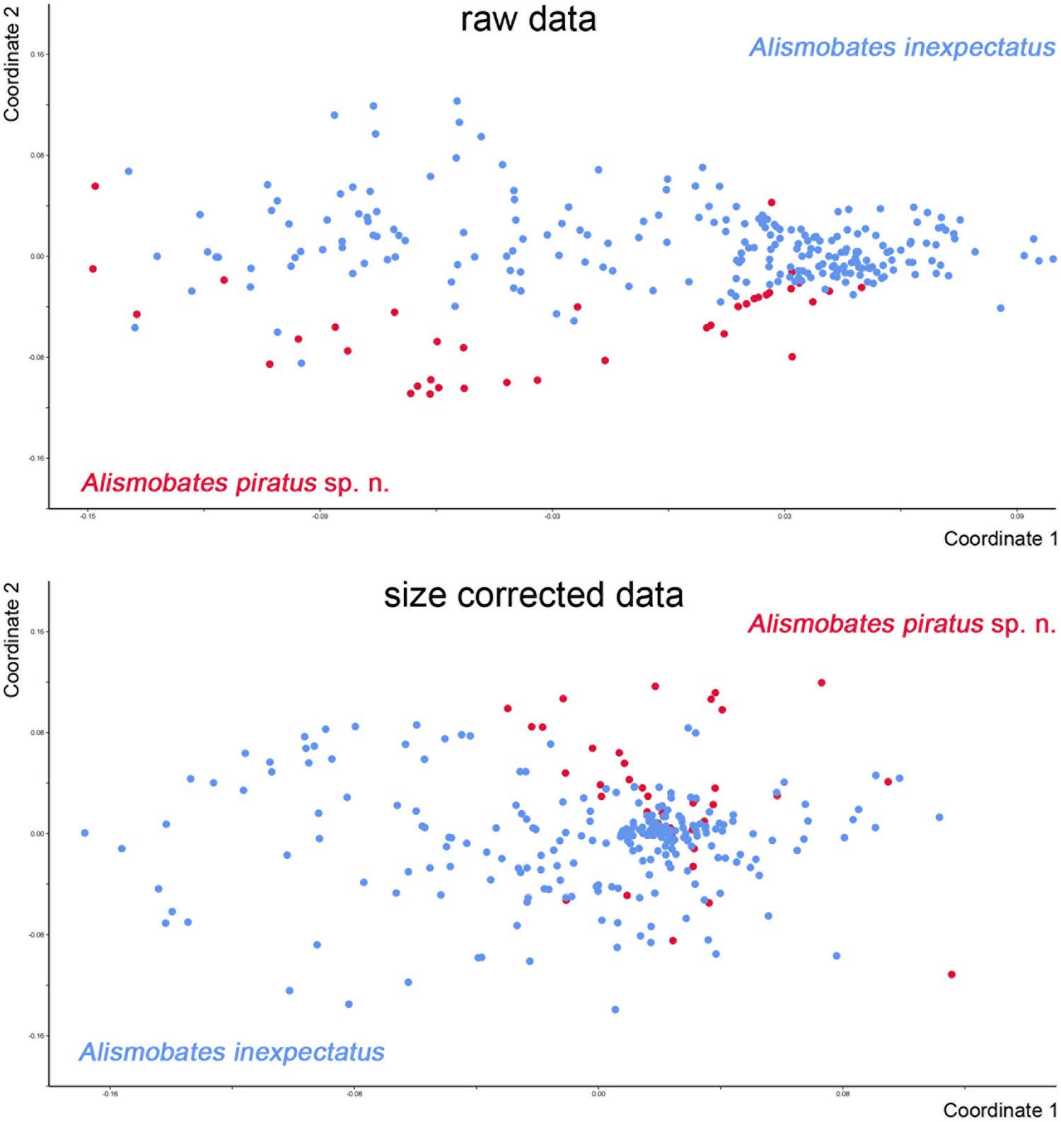
Graph showing results of Non-metric Multidimensional Scaling (NMDS) performed on raw and size-corrected morphometric data of the two *Alismobates* species

NMDS performed on raw and size corrected data resulted in two clusters showing a partial overlap (Fig. 7). Both clusters could be distinguished with a stress of 0.3759 and 0.4273 respectively. Linear discriminant analysis (LDA) shows a clearer separation between both species and could correctly classify 96.3% of the specimens using raw and size corrected data.

#### Intraspecific variation

Univariate comparison of populations of *A. inexpectatus* from the Northern Caribbean, Central America and the Antilles revealed highly significant differences in 10 out of 16 variables at least between two of the populations (Table 4). Only two characters showed no significant differences at all among all populations and these were the anterior notogastral width *nwc*_*1*_, and the distance between camerostome *dcg*. The Northern Caribbean population diverges the most and is by trend the largest, showing the highest mean values for nearly each variable.

**Table 4:** Univariate statistics for three geographic groups of *Alismobates inexpectatus* (Northern = Bermuda, Florida and Bahamas; Central America = Costa Rica and Panama; Antilles = Barbados and Dominican Republic). Range (mean ± standard deviation) of each measured variable given in µm. KW – Kruskal–Wallis Test, *0.01 < p < 0.05, **0.001 < p < 0.01, ***p < 0.001; MWU – Mann–Whitney U test,—= no significant difference, letter indicates significant difference, a = Northern Caribbean vs. Central America, b = Northern Caribbean vs. Antilles, c = Central America vs. Antilles

	Northern Caribbean (n = 156)	Central America (n = 59)	Antilles (n = 17)	KW	MWU
*bl*	344–406 (375 ± 15.2)	338–388 (361 ± 10.6)	344–377 (361 ± 7.8)	***	a, b
*dpc*	102–120 (111 ± 3.7)	99–114 (108 ± 3.7)	99–111 (107 ± 3.6)	***	a, b
*dPtI*	151–175 (164 ± 5.3)	154–169 (161 ± 3.3)	145–166 (157 ± 4.9)	***	a, b, c
*db*	92–117 (105 ± 4.5)	95–117 (105 ± 3.7)	99–105 (102 ± 2.3)	**	b, c
*ll*	52–92 (72 ± 8)	55–83 (69 ± 5.9)	59–86 (73 ± 6.5)	**	a, c
*nwc*_*1*_	157–225 (190 ± 13.1)	160–219 (187 ± 12.6)	169–203 (184 ± 10)	-	-
*nw*_*da*_	215–274 (246 ± 13.3)	200–249 (230 ± 9.9)	215–246 (230 ± 8.7)	***	a, b
*nw*_*dm*_	222–274 (247 ± 12.9)	200–249 (238 ± 9.3)	222–240 (230 ± 5.9)	***	a, b
*cl*	92–111 (101 ± 4.6)	91–105 (99 ± 4.1)	95–105 (100 ± 2.6)	*	a
*cw*	71–86 (77 ± 2.4)	71–83 (76 ± 2.4)	74–77 (76 ± 1.4)	*	a
*dcg*	74–92 (83 ± 4.2)	77–92 (84 ± 3.8)	71–90 (83 ± 5.3)	-	-
*dac3*	129–151 (136 ± 4.2)	114–142 (135 ± 4.6)	123–135 (131 ± 3.5)	***	b, c
*gl*	49–71 (62 ± 5)	49–65 (58 ± 5.1)	49–68 (57 ± 6.1)	***	a, b
*gw*	66–89 (79 ± 6.1)	65–83 (74 ± 5.3)	65–86 (73 ± 5.2)	***	a, b
*al*	80–102 (90 ± 4.3)	78–92 (87 ± 2.7)	80–89 (85 ± 2.9)	***	a, b
*aw*	71–95 (80 ± 4.3)	68–83 (76 ± 2.8)	71–83 (76 ± 3.1)	***	a, b

Variation within *Alismobates piratus* sp. n. is basically lower, there are only two variables, the body length *bl* and the anal length *al*, showing highly significant differences among the populations from the Dominican Republic, Curaçao, Guadeloupe and Barbados (Table 5). There are four other variables showing significantly different values but nine out of 16 variables show no significant deviations at all. Mann–Whitney-U test shows that the population from Curaçao is responsible for most of the found significant differences.

**Table 5:** Univariate statistics for four populations of *Alismobates piratus* sp. n. Range (mean ± standard deviation) of each measured variable given in µm. KW – Kruskal–Wallis Test, *0.01 < p < 0.05, **0.001 < p < 0.01, ***p < 0.001; MWU – Mann–Whitney U test,—= no significant difference, letter indicates significant difference, a = Dominican Rep. vs. Curaçao, b = Dominican Rep. vs. Guadeloupe, c = Dominican Rep. vs. Barbados, d = Curaçao vs. Guadeloupe, e = Curaçao vs. Barbados, f = Guadeloupe vs. Barbados

	Dominican Republic (n = 18)	Curaçao (n = 14)	Guadeloupe (n = 5)	Barbados (n = 10)	KW	MWU
*bl*	320–385 (347 ± 21.4)	363–391 (377 ± 9.4)	351–370 (362 ± 7.4)	338–369 (355 ± 12.5)	***	a, e
*dpc*	95–108 (101 ± 4.1)	95–108 (101 ± 3.3)	95–105 (102 ± 4.1)	95–108 (102 ± 3.8)	-	-
*dPtI*	149–163 (154 ± 4.7)	148–160 (152 ± 4.1)	151–154 (152 ± 1.6)	145–160 (152 ± 5.3)	-	-
*db*	95–108 (100 ± 4.4)	95–109 (101 ± 4.5)	100–108 (104 ± 3.1)	92–105 (101 ± 4.6)	-	-
*ll*	52–95 (75 ± 12.3)	77–99 (86 ± 6.1)	77–89 (83 ± 4.5)	68–89 (78 ± 6.0)	**	e
*nwc*_*1*_	166–203 (186 ± 14.4)	179–212 (194 ± 11.2)	182–194 (187 ± 4.4)	166–200 (185 ± 11.1)	-	-
*nw*_*da*_	206–249 (228 ± 14.8)	225–255 (240 ± 9.6)	229–240 (234 ± 4.7)	215–240 (228 ± 8)	*	e
*nw*_*dm*_	204–246 (225 ± 13.3)	222–255 (240 ± 9.3)	225–234 (230 ± 4.5)	200–237 (223 ± 11.4)	**	a, e
*cl*	92–108 (100 ± 4.6)	99–108 (104 ± 2.9)	102–105 (104 ± 1.6)	92–105 (100 ± 4.4)	*	-
*cw*	74–77 (75 ± 1.5)	71–77 (75 ± 1.9)	72–77 (74 ± 1.8)	71–77 (74 ± 2.4)	-	-
*dcg*	71–86 (76 ± 4.3)	71–89 (80 ± 4.8)	71–80 (75 ± 3.3)	74–86 (79 ± 4.3)	*	-
*dac3*	121–135 (127 ± 4.4)	123–132 (127 ± 2.8)	123–132 (128 ± 3.4)	120–135 (129 ± 5.2)	-	-
*gl*	49–68 (56 ± 7.1)	51–65 (58 ± 5.2)	55–67 (61 ± 4.9)	49–68 (57 ± 6.7)	-	-
*gw*	65–81 (70 ± 6.3)	65–80 (73 ± 6.2)	66–77 (73 ± 4.2)	63–80 (71 ± 6.4)	-	-
*al*	74–92 (80 ± 5.8)	86–95 (90 ± 2.5)	83–92 (87 ± 3.5)	80–92 (85 ± 3.8)	***	a, e
*aw*	71–77 (74 ± 2.2)	71–80 (75 ± 3.3)	68–75 (72 ± 2.8)	68–78 (74 ± 3.3)	-	-

**Fig. 8:**
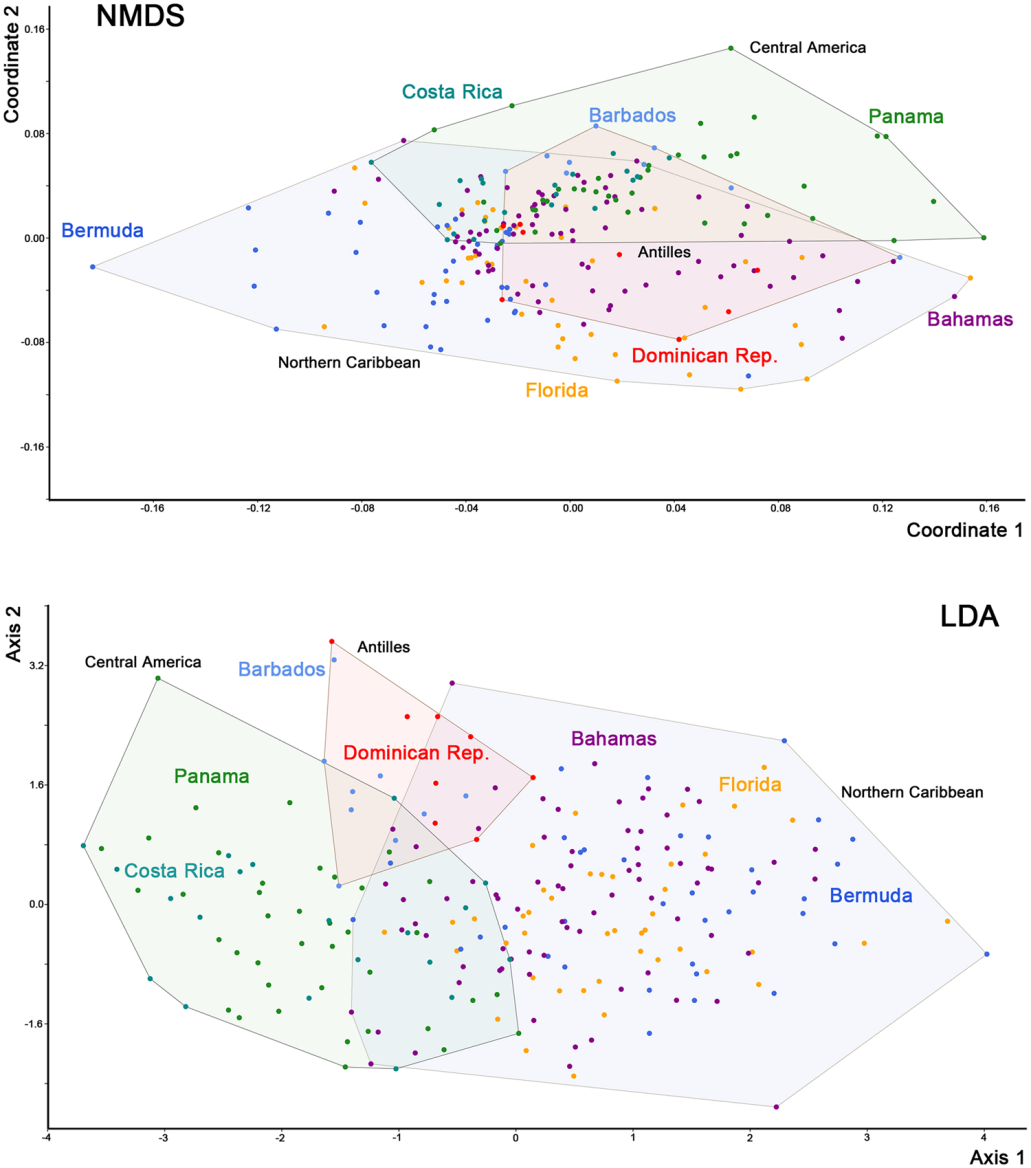
Graph highlighting results of Non-metric Multidimensional Scaling (NMDS) and Linear Discriminant Analysis (LDA) performed on size corrected data of seven Caribbean *Alismobates inexpectatus* populations. Slightly coloured and framed areas refer to geographic regions: Northern Caribbean = Bermuda, Florida, Bahamas; Antilles = Barbados, Dominican Rep.; Central America = Costa Rica, Panama

Multivariate analyses of *A. inexpectatus* populations resulted generally in large overlaps but with a distinct trend for diversification. NMDS on size corrected data resulted in three overlapping clusters (Fig. 8) that could be distinguished with a stress of 0.37. LDA showed better results with fewer overlaps and with 83.19% correctly classified specimens using size corrected data. The Northern Caribbean population shows the largest cluster with most variation in both analyses.

**Fig. 9:**
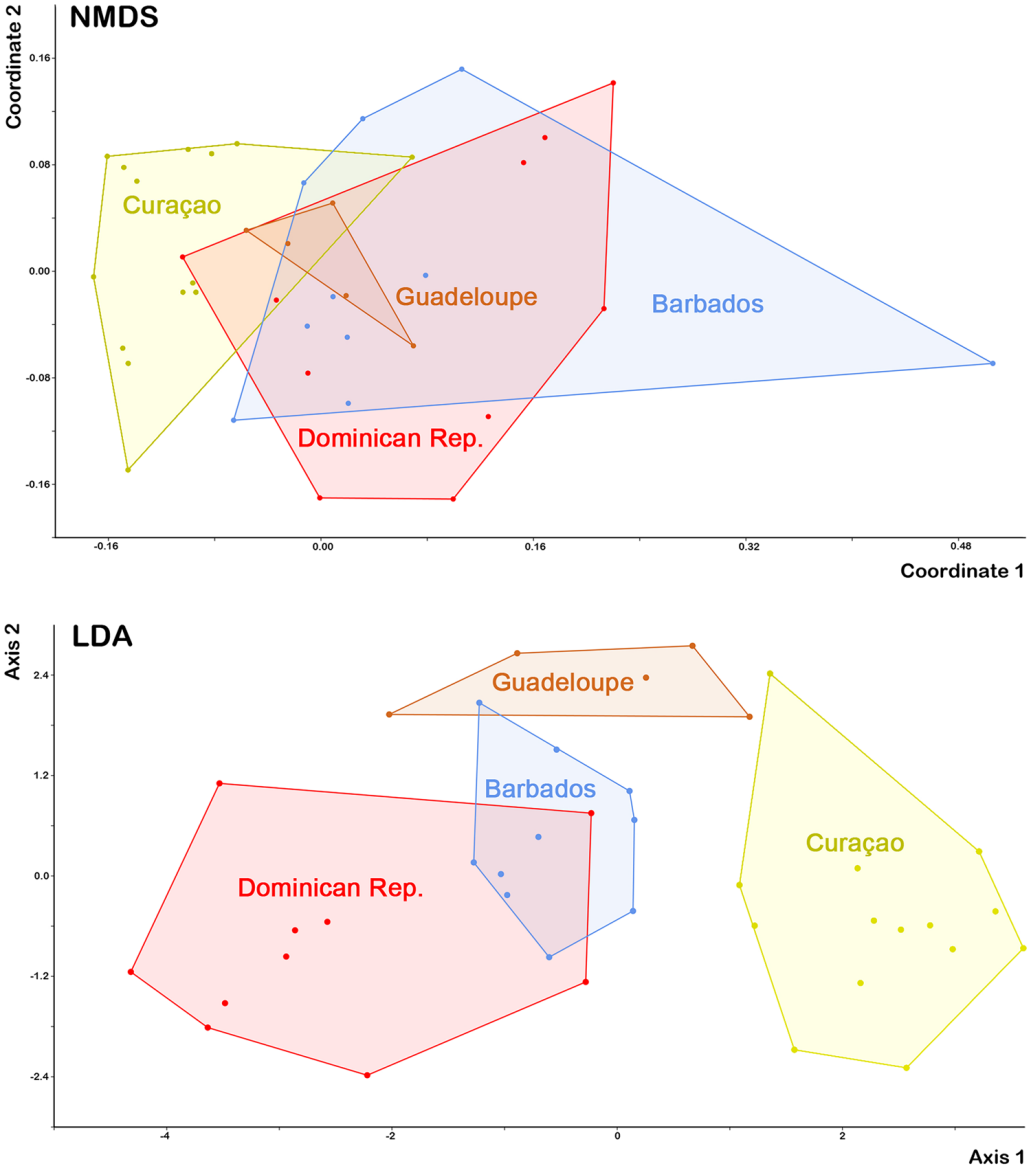
Graph showing results of Non-metric Multidimensional Scaling (NMDS) and Linear Discriminant Analysis (LDA) performed on size corrected data of four Caribbean *Alismobates piratus* sp. n. populations

NMDS performed on size corrected data of *A. piratus* sp. n. resulted in four clusters, with the population from Curaçao showing only minor overlaps, whereas the other populations from the Dominican Republic, Guadeloupe and Barbados strongly overlap (Fig. 9). Populations could be separated with a stress of 1.9. When these populations are subjected to LDA, populations from Guadeloupe and Curaçao form separate clusters and the Dominican Republic and Barbados still show slight overlaps. 93.2% of the specimens were correctly classified using size corrected data.

## Discussion

### Cryptic diversity

All collected *Alismobates* specimens from across the Caribbean show conformity in their morphology and though morphometric data indicates certain differentiation in size and shape, all measured variables show overlaps in their ranges leaving no distinct trait for separation of any group. Molecular genetic data, on the other hand, show distinct structures and strong diversification. Species delimitation analyses support the existence of more than 10 different Caribbean *Alismobates* species, but tree based methods are known to often oversplit species (e.g. Dellicour & Flot, 2018). The present dataset is highly structured due to the geographic setting with many isolated islands and species delimitation approaches apparently identify somewhat divergent singletons as distinct species. It is reasonable not to assume so many species. Divergence on the *COI* and *18S* rRNA gene fragments clearly exceeds intraspecific variation for at least two distinct lineages and thus reveal the existence of just a single genetically distinct species, namely *Alismobates piratus* sp. n. The contrast between morphology and genetic sequences renders *A. piratus* sp. n. a so called ‘cryptic species’, which is defined as a species that is difficult to distinguish with traditional morphology-based taxonomic methods (Knowlton, 1993). Despite the identical phenotype, the present results clearly show that *A. inexpectatus* and *A. piratus* sp. n. are separately evolving metapopulation lineages, which makes them species in the sense of the unified species concept (De Queiroz, 2007). Moreover, individuals of both supposed species were found syntopically at the exact same location and did not show any signs of hybridization, consequently they show reproductive isolation and thus are also species according to the biological species concept (Mayr, 1940) (Table 6).

So why do both species still show the same morphology? Extreme and homogeneous environments may cause stabilizing selection that reduces morphological change usually accompanying speciation (Bickford et al., 2007). The intertidal zone represents such an extreme environment and thus may be responsible for the observed morphological stasis. Cryptic diversity was also reported in several other Caribbean intertidal oribatid mite taxa, i.e. *Carinozetes*, *Fortuynia* and *Thalassozetes*, (Pfingstl et al., 2019a, b, 2021, 2022) which confirms a correlation between the intertidal environment and morphological stasis.

However, the reasons for cryptic speciation in these Caribbean intertidal oribatid mite species may differ. In the cryptically diverse *Carinozetes*, neither the found biogeographic pattern nor the ecological needs could explain the genetic diversification of the lineages (Pfingstl et al., 2019a, b), and thus the cause for speciation remains unknown in this case. In the cryptic *Thalassozetes*, on the other hand, vicariance is most likely the reason, because nearly all species are clear island endemics showing very poor dispersal ability (Pfingstl et al., 2021). Vicariance is also supposed to be responsible for the diversification of the northern Caribbean *Fortuynia atlantica* and the Lesser Antillean *F. antillea* Pfingstl et al., 2022 (Pfingstl et al., 2022). The present *Alismobates* species show a somewhat similar pattern with *A. inexpectatus* being more distributed in the northern and western parts of the Caribbean, and *A. piratus* sp. n. being restricted to Hispaniola and the Lesser Antilles, whereas distributional ranges are overlapping in the latter areas. At some point in geological history, there may have been a biogeographic barrier between more northern and Antillean ancestral populations of Caribbean *Fortuynia* and *Alismobates*, which caused allopatric speciation. Sometime later the barrier might have vanished and species expanded distribution ranges resulting in the observed sympatric patterns, at least in *Alismobates*. However, based on the present results, we are not able to allocate this suggested geographic barrier to any geological event or formation and further studies are needed to identify the reason for the speciation of the Caribbean *Alismobates* species.

### Phylo-and biogeographic patterns

Haplotype network analysis of mitochondrial *COI* sequences show that *Alismobates piratus* sp. n. populations from different Antillean islands exhibit a distinct pattern with strong differentiation among them. Accordingly, there was no recent gene flow between populations and they have been separated for a long time. Most of the Lesser Antilles are relatively recent volcanic islands with an age of less than 10 million years (Iturralde-Vinent & MacPhee, 1999), the oldest rock of Guadeloupe, for example, is dated 4.7 million years (Maury et al., 1990). Presently, there is no reliable substitution rate inferred for the *COI* gene of mites, but using the general arthropod substitution rate of 1–1.15%/my (DeSalle et al., 1987) would result in a separation of approx. 2.7–3 million years among the *A. piratus* sp. n. populations. This date would coincide with the emergence of at least some of the Lesser Antilles, but as said before, the substitution rate is not reliable and thus we cannot assign the diversification of the populations to a certain time period. However, it is assumable that the *A. piratus* sp. n. populations from the Lesser Antilles are derived from stocks in Hispaniola because this Greater Antillean Island is much older with an approximate age of 40 million years (Iturralde-Vinent & MacPhee, 1999) and there is a recent population present on this landmass. Certain arachnids show strong diversification within the islands of Jamaica and Hispaniola and basically a higher diversity in the Greater Antilles relative to the Lesser Antilles (Crews & Gillespie, 2010), indicating that these older landmasses are centers of species diversity within the Caribbean. This could also apply to *Alismobates* with the ancestral species having diversified on the Greater Antilles and descendants having subsequently colonized newly emerged islands. Nevertheless, further comprehensive sampling and studies, including more islands of the Greater and Lesser Antilles, are necessary to verify this assumption.

The *A. inexpectatus* populations, on the other hand, show varying haplotype patterns in different Caribbean regions. First, the populations from the Dominican Republic/Hispaniola and Barbados exhibit large divergence, which is well in accordance with the pattern found in *A. piratus* sp. n. populations from the same areas. Second, the Central American populations show deep splits and distinct structures although all individuals were collected on the Bocas del Toro archipelago in a range of less than 30 km. The Central American Landmass has been subject to tremendous changes during the last 40 million years and the Panamanian Isthmus was finally completed about 2.5–2.3 million years ago (e.g. Iturralde-Vinent, 2006). The latter event led to enormous environmental changes, which may have caused the separation of *A. inexpectatus* populations in this area. Moreover, the closure of the Central American land bridge caused a deflection of the circumtropical current and gave birth to the Caribbean current, flowing along the South and Central American coastline (e.g. Iturralde-Vinent & MacPhee, 1999). Intertidal mites are supposed to be dispersed over long distances by drifting on ocean currents (Pfingstl, 2013; Schatz, 1991), consequently populations from more eastern South American coastal areas may have occasionally colonized the Bocas del Toro archipelago via the Caribbean current. Thus, the distinct heterogenous genetic structure found in Panamanian *A. inexpectatus* populations may be a product of the dynamic geological and environmental history of the Central American Isthmus during the Pliocene. Third, northern Caribbean populations show less distinct structuring in the *COI* gene sequences and haplotype network analyses indicate a common origin and subsequent expansion events on the Bahamas and the Bermudas. The Bahamas platform has been a stable carbonate block since the Cretaceous but emerging islands have only been created by accumulation and sedimentation during the Pleisto- and Holocene (Carew & Mylroie, 1997). It seems that New Providence Island has been colonized by a few individuals sometime during this period and then populations have expanded and slowly diversified over the island. A single haplotype is shared by northeastern and southeastern populations indicating that initial colonization may have happened in the eastern parts of this landmass. A similar haplotype pattern is found in Bermudian populations. Bermuda is a small remote oceanic landmass that emerged from the sea ca. 900,000 years ago (Thomas, 2004). The present data indicates that it was colonized early in this period and then populations slowly diversified over the island, whereas a single haplotype is still found in four out of the five sampled locations. Another interesting pattern is found in the specimens from the Florida Keys; there are two groups of haplotypes that are only distantly related, which indicates that one of the groups most likely originates from a different area in the Caribbean.

The genetic landscape analysis of all Caribbean *Alismobates* shows high divergence in the Western Atlantic and parts of the Caribbean Sea, like the Colombia and the Caribbean Basin, and less genetic divergence in the Western and northern Caribbean areas (see Fig. 4). The former areas represent vast stretches of open ocean and it makes absolute sense that these areas are rather effective barriers for the dispersal of tiny mites. The latter areas, on the other hand, mostly represent continuous continental coastlines which may allow gene flow along the coast, at least over short distances. However, there is another important factor that seems to strongly contribute to the observed pattern, namely the Gulf Stream. This strong current flows along the Central American coast, passes Florida and the Bahamas and finally runs past Bermuda (see Fig. 2). Considering long distance transport of mites drifting on the ocean’s surface, the Gulf Stream could explain the strong connectivity between Central America and the Northern Caribbean, as well as between the Northern Caribbean and the far remote landmass of Bermuda. A strong link and gene flow between Central America and North America was also shown in the intertidal oribatid mite *Thalassozetes balboa* Pfingstl et al., 2019a, b, because populations from Panama and Florida share several haplotypes (Pfingstl et al., 2021). Other studies (Pfingstl, 2013; Schatz & Schuster, 2012) already hypothesized that the Gulf Stream may be responsible for the colonization of Bermuda by several oribatid mites from the Northern Caribbean, and the recent study on the Caribbean intertidal mite *F. atlantica* (Pfingstl et al., 2022) supported this theory for the first time with molecular genetic data. The present results further corroborate a link between the Northern Caribbean and Bermuda, and highlight the important role of the Gulf Stream for the dispersal of intertidal oribatid mites.

### Electronic supplementary material

Below is the link to the electronic supplementary material.

Supplementary file1 (PDF 1040 KB)

## Data Availability

All sequences obtained from this study were deposited in GenBank (www.ncbi.nlm.nih.gov/genbank) under the accession numbers OR358561-OR358798 and OR360272-OR360328.

## Appendix

**Table 6:** GenBank accession numbers for *COI* and *18S* rRNA sequences comprising all specimens included in genetic investigations

					**GenBank Accession**	
**Country**	**Location**	**Sample ID**	**Species**	**Coordinates**	**COI**	**18S rRNA**
Barbados	Bathsheba	BA_13_01	*A. inexpectatus*	13.213011, -59.520318	OR358561	
		BA_13_02			OR358562	
		BA_13_03			OR358563	
		BA_13_05			OR358564	
	Bathsheba	BA_15_11		13.21393, -59.521825	OR358564	
	Oistins	BA_22_01	*A. piratus*	13.062537, -59.541903	OR358566	OR360272
		BA_22_02			OR358567	OR360273
		BA_22_04			OR358568	OR360274
		BA_22_06			OR358569	OR360275
	Bathsheba	BA_28_01	*A. inexpectatus*	13.062537, -59.541903	ON239341	ON243686
Bermuda	Whalebone Bay	BD_07_01		32.365424, -64.713527	OR358570	OR360276
		BD_07_02			OR358571	
		BD_07_03			OR358572	
		BD_07_04			OR358573	
		BD_07_05			OR358574	
		BD_07_06			OR358575	
		BD_07_07			OR358576	
		BD_07_09			OR358577	OR360277
		BD_07_10			OR358578	
		BD_07_11			OR358579	
		BD_07_12			OR358580	
		BD_07_13			OR358580	
		BD_07_14			OR358582	
		BD_07_15			OR358583	OR360278
	Whalebone Bay	BD_08_02	*A. inexpectatus*	32.365424, -64.713527	ON239352	ON243685
	Tobacco Bay	BD_11_01		32.389109, -64.678894	OR358584	
		BD_11_02			OR358585	OR360279
		BD_11_03			OR358586	OR360280
		BD_11_04			OR358587	
		BD_11_05			OR358588	
		BD_11_06			OR358589	
		BD_11_07			OR358590	
		BD_11_08			OR358591	
		BD_11_09			OR358592	OR360281
		BD_11_10			OR358593	
	Hungry Bay	BD_21_01		32.290249, -64.759893	OR358594	OR360282
		BD_21_02			OR358595	
		BD_21_03			OR358596	
		BD_21_04			OR358597	
		BD_21_05			OR358598	
		BD_21_07			OR358599	
		BD_21_08			OR358600	
		BD_21_09			OR358601	
		BD_21_10			OR358602	
		BD_21_11			OR358603	
		BD_21_12			OR358604	
		BD_21_13			OR358605	
		BD_21_14			OR358606	
		BD_21_15			OR358607	
	Coney Island	BD_25_02		32.357888, -64.716516	OR358608	OR360283
		BD_25_04			OR358609	
		BD_25_05			OR358610	OR360284
	Whalebone Bay	BD_36_01		32.365424, -64.713527	OR358611	
		BD_36_02			OR358612	OR360285
		BD_36_03	*A. inexpectatus*		OR358613	
		BD_36_04			OR358614	
		BD_36_05			OR358615	
		BD_36_06			OR358616	OR360285
		BD_36_07			OR358617	
		BD_36_08			OR358618	
		BD_36_09			OR358619	
	Cooper’s Island	BD_40_01		32.346514, -64.652784	OR358620	
		BD_40_02			OR358621	
		BD_40_03			OR358622	
		BD_40_04			OR358623	
		BD_40_05			OR358624	
		BD_40_06			OR358625	
		BD_40_07			OR358626	
		BD_40_08			OR358627	
		BD_40_09			OR358628	
		BD_40_10			OR358629	
Bahamas	Paradise Island	BH_03_01		25.085983, -77.299663	OR358630	
		BH_03_02			OR358631	
		BH_03_03			OR358632	
		BH_03_04			OR358633	
		BH_03_05			OR358634	
		BH_03_06			OR358635	
		BH_03_07			OR358636	
		BH_03_08			OR358637	
		BH_03_09			OR358638	
		BH_03_10			OR358639	
	Love Beach	BH_08_01		25.062984, -77.491670	OR358640	OR360287
		BH_08_02			OR358641	OR360288
		BH_08_03			OR358642	
		BH_08_04	*A. inexpectatus*		OR358643	OR360289
		BH_08_05			OR358644	
		BH_08_06			OR358645	OR360290
		BH_08_07			OR358646	
		BH_08_08			OR358647	
		BH_08_09			OR358648	
		BH_08_10			OR358649	
	Compass Point	BH_10_01		25.065252, -77.470981	OR358650	OR360291
		BH_10_02			OR358651	OR360292
		BH_10_03			OR358652	
		BH_10_04			OR358653	
		BH_10_06			OR358654	
	South Beach	BH_16_01		25.001339, -77.350229	OR358655	
		BH_16_02			OR358656	OR360293
		BH_16_03			OR358657	
		BH_16_04			OR358658	
		BH_16_05			OR358659	
		BH_16_06			OR358660	
		BH_16_07			OR358661	
		BH_16_08			OR358662	
		BH_16_09			OR358663	OR360294
		BH_16_10			OR358664	
		BH_16_11			OR358665	
		BH_16_12			OR358666	OR360295
		BH_16_13			OR358667	
		BH_16_14			OR358668	
	South Beach	BH_17_01		25.001339, -77.350229	OR358669	OR360296
		BH_17_02			OR358670	
		BH_17_03			OR358671	OR360297
		BH_17_04			OR358672	
		BH_17_05	*A. inexpectatus*		OR358673	OR360298
	Jaws Beach	BH_19_01		25.018155, -77.546636	OR358674	
		BH_19_02			OR358675	OR360299
		BH_19_03			OR358676	
		BH_19_04			OR358677	
		BH_19_05			OR358678	
		BH_19_06			OR358679	
		BH_19_07			OR358680	
		BH_19_08			OR358681	
		BH_19_09			OR358682	OR360300
		BH_19_10			OR358683	
		BH_19_11			OR358684	
		BH_19_12			OR358685	OR360301
		BH_19_13			OR358686	
		BH_19_14			OR358687	
		BH_19_15			OR358688	
	Montagu Beach	BH_21_01		25.073943, -77.307127	OR358689	
		BH_21_02			OR358690	
		BH_21_03			OR358691	
		BH_21_04			OR358692	
		BH_21_05			OR358693	
Curaçao	Boca Ascención	CU_15_01	*A. piratus*	12.273242, -69.052882	OR358694	OR360302
		CU_15_03			OR358695	
		CU_15_04			OR358696	
		CU_15_05			OR358697	OR360303
		CU_15_06			OR358698	
		CU_15_07			OR358699	
		CU_15_08			OR358700	
		CU_15_09			OR358701	
		CU_15_10			OR358702	
		CU_15_11	*A. piratus*		OR358703	OR360304
		CU_15_13			OR358704	
		CU_15_14			OR358705	
		CU_15_15			OR358706	
Dominican Rep	Boca Chica	DR_03_01	*A. inexpectatus*	18.447819, -69.620430	OR358707	
		DR_03_02			OR358708	OR360305
		DR_03_03			OR358709	
		DR_03_04			OR358710	OR360306
		DR_03_05			OR358711	
		DR_03_06			OR358712	OR360307
		DR_03_07			OR358713	
	Samaná	DR_10_01	*A. piratus*	19.201034, -69.324964	OR358714	OR360308
		DR_10_02	*A. inexpectatus*		OR358715	
		DR_10_04			OR358716	OR360309
		DR_10_05			OR358717	OR360310
		DR_10_06			OR358718	
		DR_10_07			OR358719	
		DR_10_08			OR358720	
		DR_10_09	*A. piratus*		OR358721	OR360311
		DR_10_10	*A. inexpectatus*		OR358722	
		DR_10_11			OR358723	
		DR_10_12			OR358724	
		DR_10_13			OR358725	OR360312
		DR_10_14			OR358726	
		DR_10_15			OR358727	
Florida	Indian Key Fill	FL_12_01		24.892094, -80.670667	OR358728	
		FL_12_03			OR358729	
		FL_12_04			OR358730	
		FL_12_05			OR358731	
		FL_12_06	*A. inexpectatus*		OR358732	
	Sombrero Beach	FL_16_01		24.691913, -81.083700	OR358733	OR360313
		FL_16_02			OR358734	
		FL_16_03			OR358735	
		FL_16_04			OR358736	
		FL_16_05			OR358737	
		FL_16_06			OR358738	
		FL_16_07			OR358739	
		FL_16_08			OR358740	
		FL_16_09			OR358741	OR360314
		FL_16_10			OR358742	
		FL_16_11			OR358743	
		FL_16_12			OR358744	
		FL_16_13			OR358745	
		FL_16_14			OR358746	OR360315
		FL_16_15			OR358747	
	Islamorada	FL_18_04		24.9378169, -80.612182	ON239397	ON243684
	Key Largo	FL_19_01		25.183048, -80.353081	OR358748	OR360316
		FL_19_02			OR358749	
		FL_19_03			OR358750	
		FL_19_04			OR358751	
		FL_19_05			OR358752	
		FL_19_06			OR358753	OR360317
		FL_19_07			OR358754	
		FL_19_09			OR358755	
		FL_19_10			OR358756	
		FL_19_11			OR358757	
		FL_19_12			OR358758	OR360318
		FL_19_13			OR358759	
		FL_19_14			OR358760	
		FL_19_15	*A. inexpectatus*		OR358761	
		FL_19_16			OR358762	
Guadeloupe	Capesterre Belle-Eau	GU_09_01	*A. piratus*	16.034611, -61.564938	OR358763	
		GU_09_02			OR358764	OR360319
		GU_09_03			OR358765	OR360320
		GU_09_04			OR358766	OR360321
		GU_09_05			OR358767	
		GU_09_06			OR358768	
		GU_09_07			OR358769	
		GU_09_08			OR358770	
		GU_09_09			OR358771	
Panama	Isla Colon	PA_39_01	*A. inexpectatus*	9.385454, -82.23524	OR358772	OR360322
		PA_39_03			OR358773	OR360323
		PA_39_04			OR358774	
		PA_39_05			OR358775	OR360324
		PA_39_06			OR358776	
		PA_39_07			OR358777	
		PA_39_08			OR358778	
		PA_39_09			OR358779	
		PA_39_10			OR358780	
		PA_39_12			OR358781	OR360325
		PA_39_13			OR358782	
		PA_39_14			OR358783	
		PA_39_15			OR358784	
	Isla Bastimentos	PA_41_01		9.344917, -82.180404	OR358785	
		PA_41_02			OR358786	
		PA_41_03			OR358787	OR360326
		PA_41_04			OR358788	
		PA_41_05			OR358789	
		PA_41_06			OR358790	
		PA_41_07	*A. inexpectatus*		OR358791	
		PA_41_08			OR358792	
		PA_41_09			OR358793	OR360327
		PA_41_10			OR358794	
		PA_41_12			OR358795	OR360328
		PA_41_13			OR358796	
		PA_41_14			OR358797	
		PA_41_15			OR358798	
**Outgroups**						
Malaysia	Pantai Pasir Hitam	A_MY_07_7	*A. pseudoreticulatus*		-	MH285696
	Datai Bay	A_MY_11_5			MH285618	-
Thailand	Nang Thong Beach	A_TH_06_1			MH285619	-
Japan	Iriomote-jima	JP50_Al_03	*A. reticulatus*		MN385067	-
	Okinawa-jima	-			-	AB818526
Barbados	Bridgetown	BA_17_12	*F. antillea*	13.079329, -59.61255	ON239338	ON243683
Bahamas	South Beach	BH_14_01	*F. atlantica*	25.001339, -77.350229	ON239366	ON243681
Florida	Islamorada	FL_18_03		24.9378169, -80.612182	ON239396	ON243678
