# Supplementary material for: The Caribbean intertidal mite Alismobates inexpectatus (Acari, Oribatida), an unexpected case of cryptic diversity?
Source: Org Divers Evol. 2023 Sep 29;23(4):811–32. doi: 10.1007/s13127-023-00624-9 (PMC10689554; doi:10.1007/s13127-023-00624-9)

The Caribbean intertidal mite *Alismobates inexpectatus* (Acari, Oribatida), an unexpected case of cryptic diversity?

Organisms, Diversity and Evolution

Tobias PFINGSTL*[1], Iris BARDEL-KAHR[1], Sylvia SCHÄFFER[1]

[1]*Institute of Biology, Karl-Franzens-University Graz, Universitätsplatz 2, 8010 Graz, Austria*

**Corresponding author.* E-mail: tobias.pfingstl@uni-graz.at

Supplementary Information

Fig S1. Bayesian Inference tree based on *18S* rRNA gene sequences of Caribbean *Alismobates* specimens.

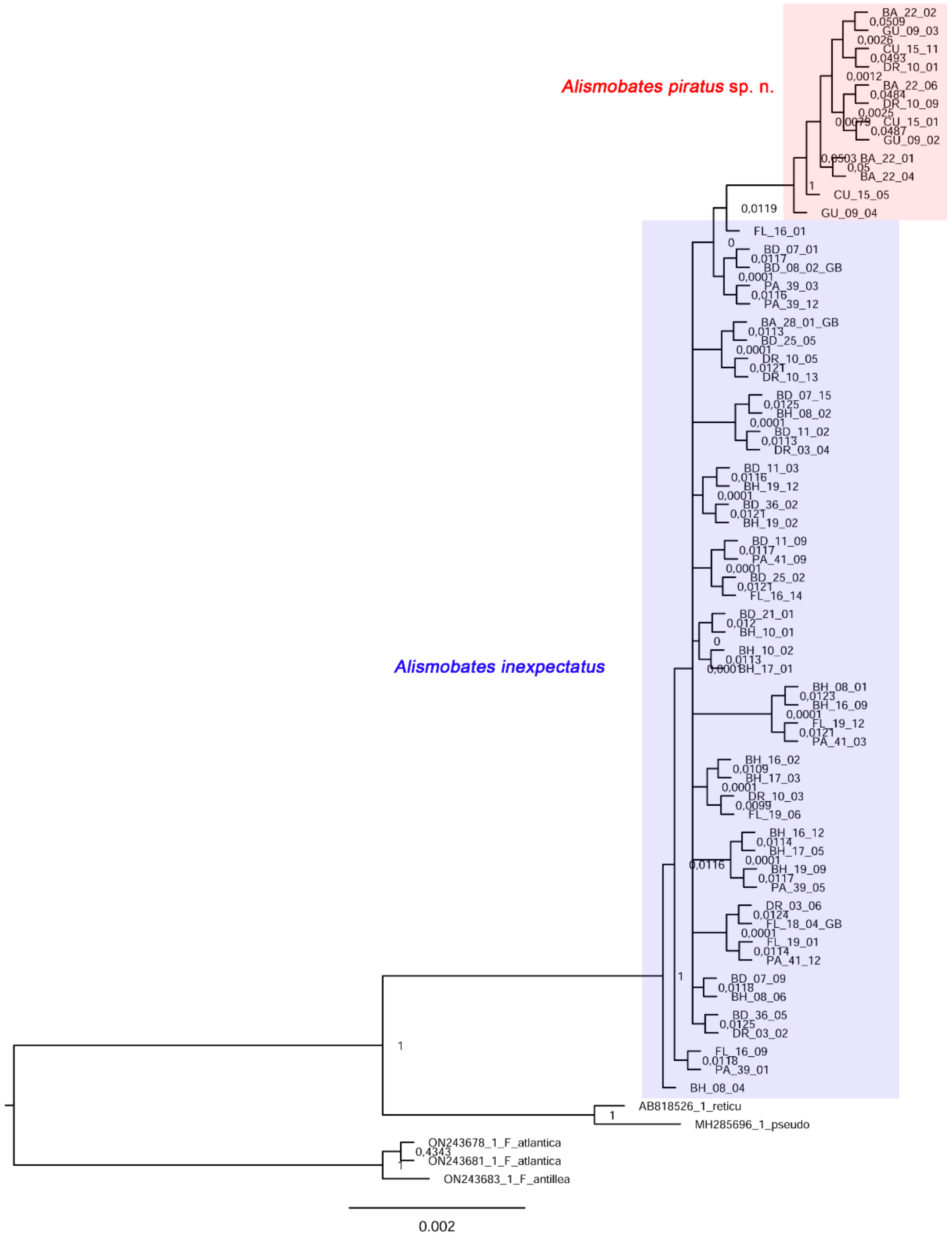

Fig S2. ML tree based on *18S* rRNA gene sequences of Caribbean *Alismobates* specimens.

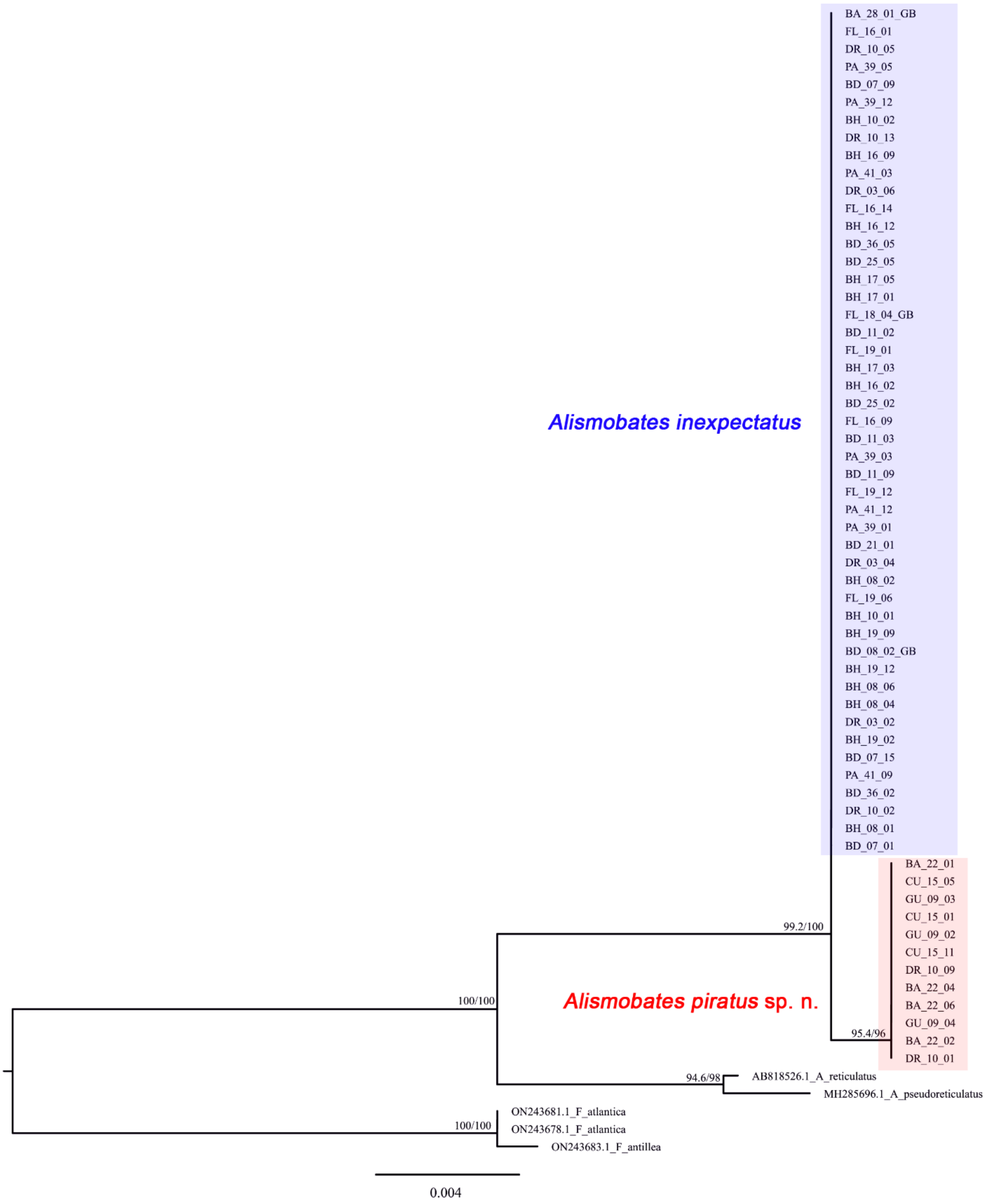

Fig S3. Bayesian Inference tree based on mitochondrial *COI* gene sequences of Caribbean *Alismobates* specimens.

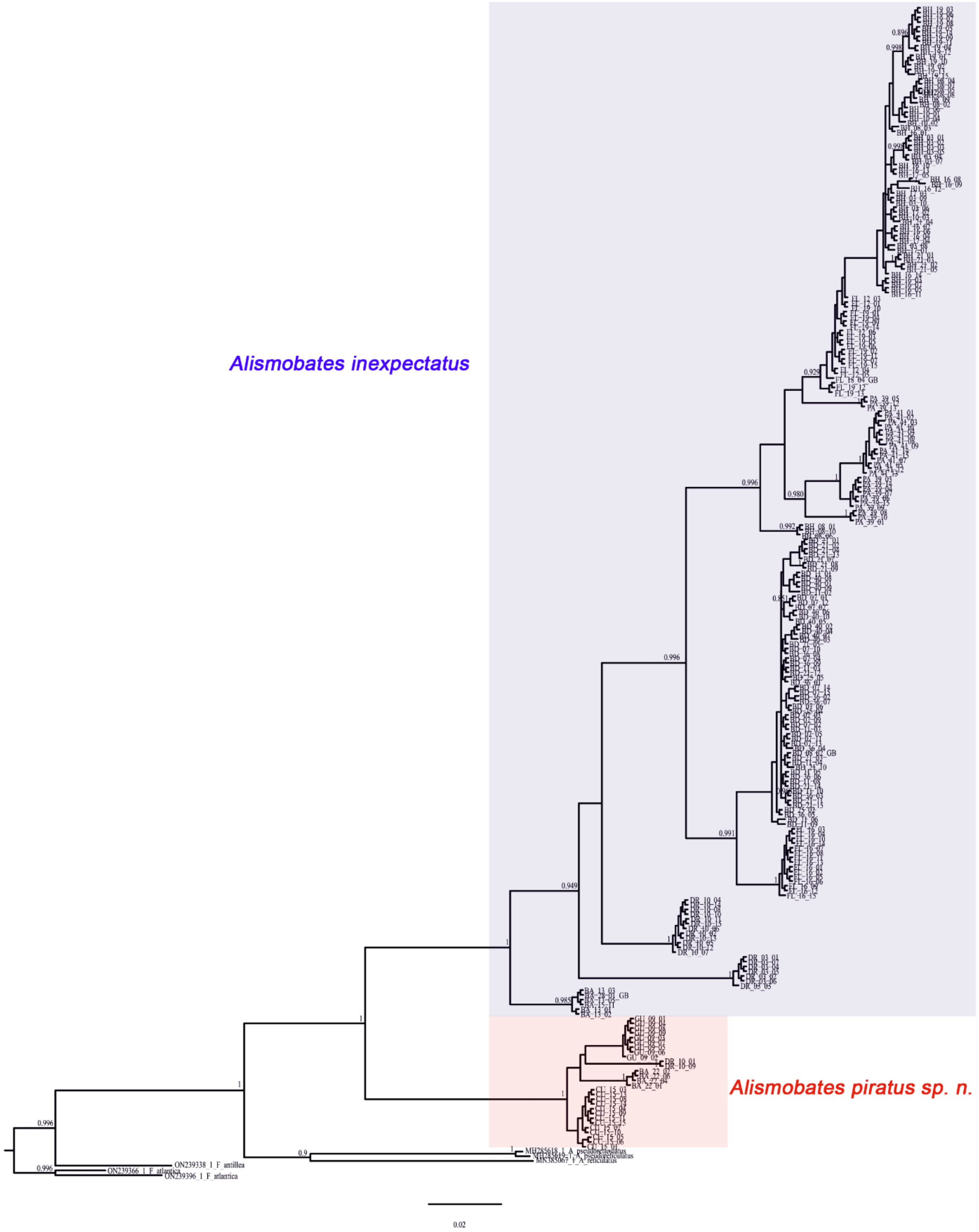

Fig S4. ML tree based on mitochondrial *COI* gene sequences of Caribbean *Alismobates* specimens.

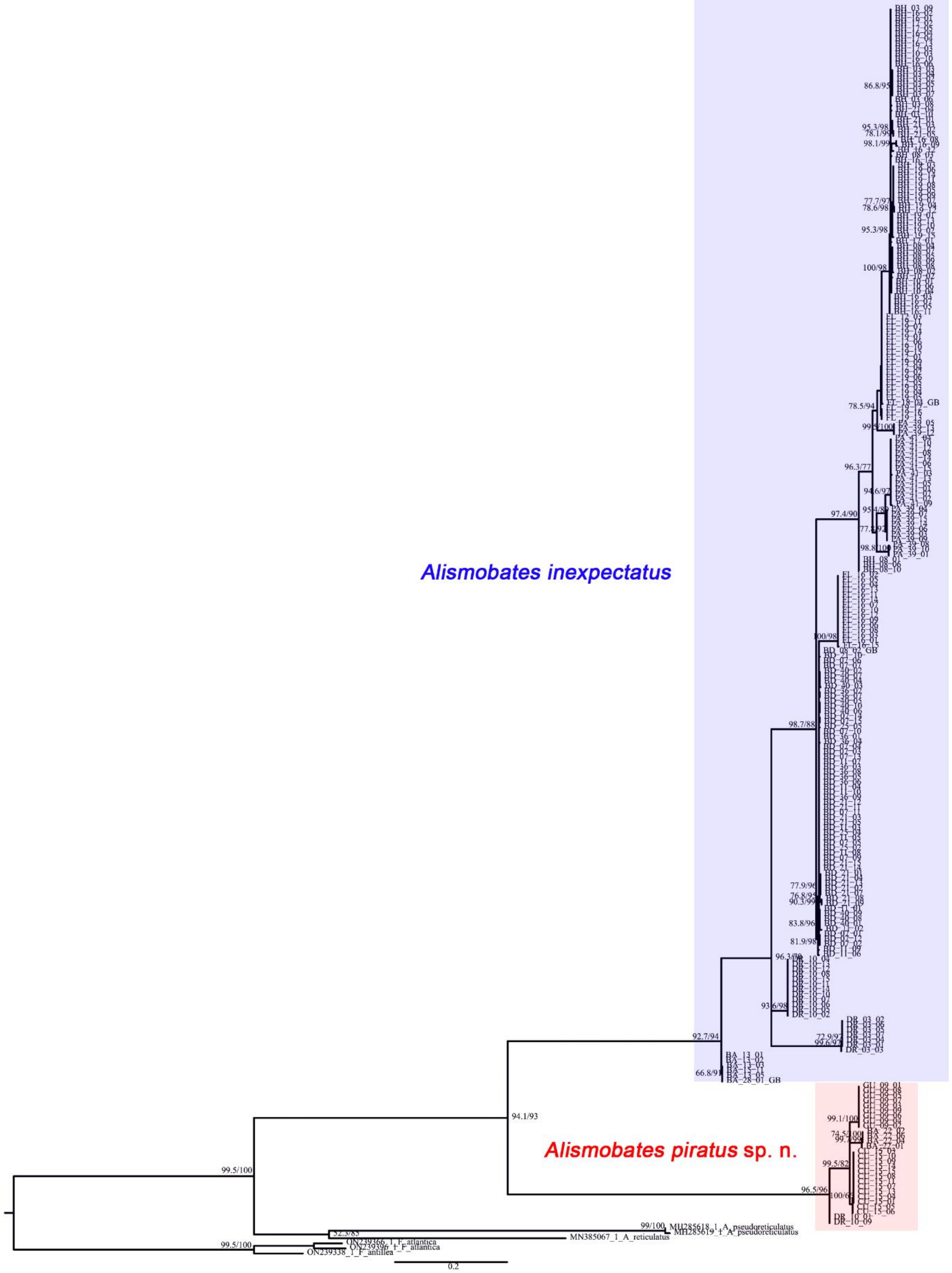

Fig S5. Species delimitation analyses bPTP and ASAP based on COI sequences. Single left column shows result of bPTP. Other columns on the right show 10 best partitions found by ASAP (far left column shows lowest score, far right column highest score).

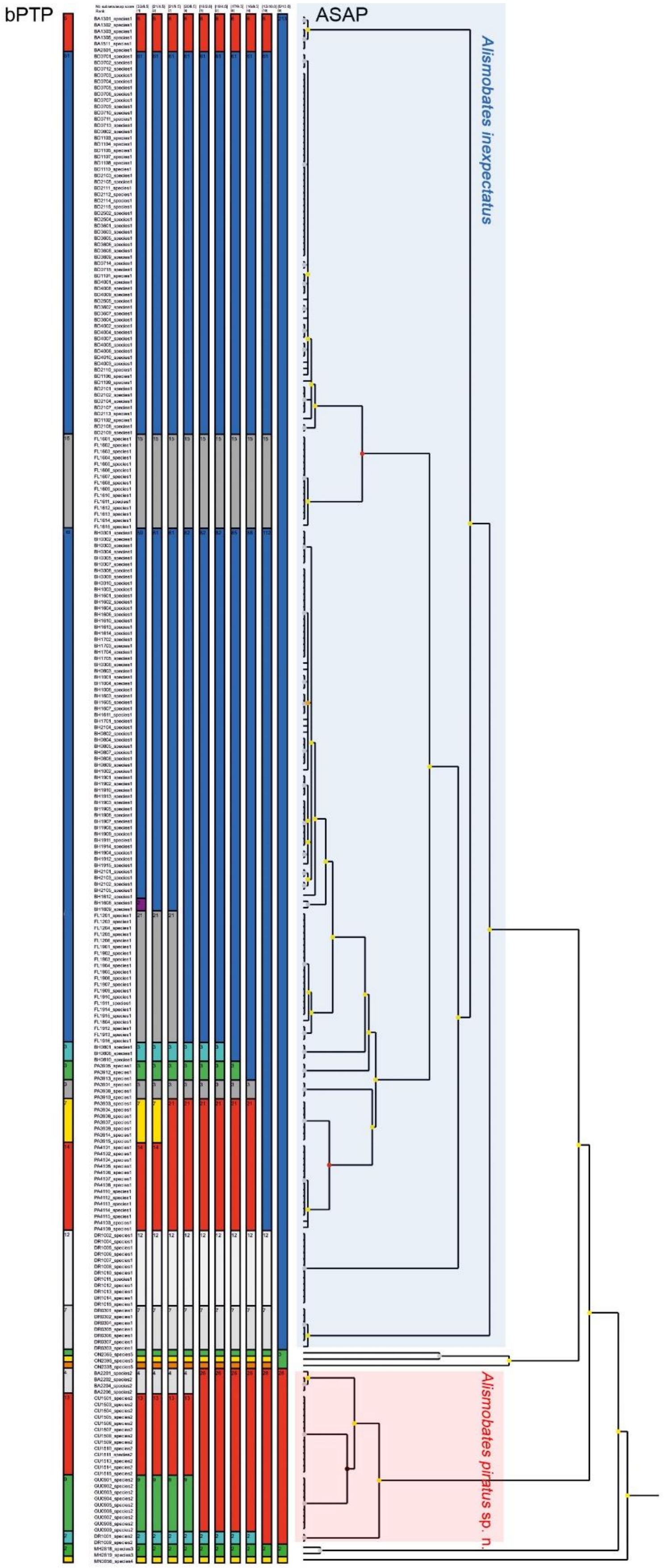

Fig S5. Photographic comparison of *Alismobates inexpectatus* and *A. piratus* sp. n. specimens from different locations (dorsal view, automatically stacked images).

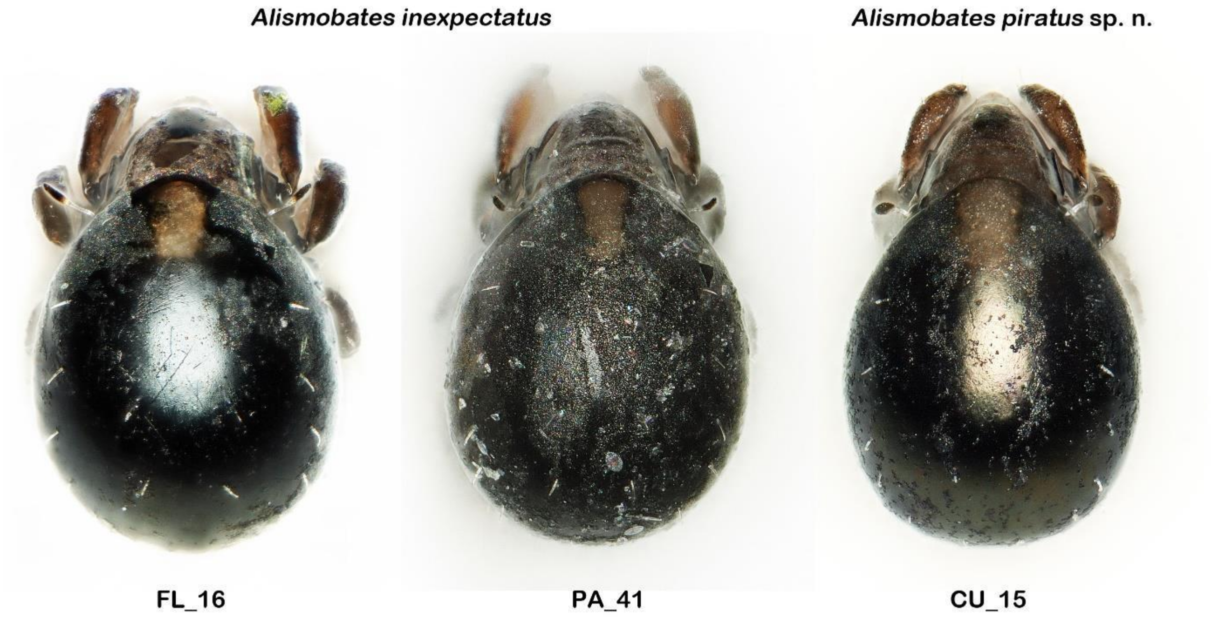

Supplement: Supplementary file 1 — Supplementary file1 (PDF 1040 KB) [file 13127_2023_624_MOESM1_ESM.pdf]
